# NMDA receptor–BK channel coupling regulates synaptic plasticity in the barrel cortex

**DOI:** 10.1073/pnas.2107026118

**Published:** 2021-08-27

**Authors:** Ricardo Gómez, Laura E. Maglio, Alberto J. Gonzalez-Hernandez, Belinda Rivero-Pérez, David Bartolomé-Martín, Teresa Giraldez

**Affiliations:** ^a^Departamento de Ciencias Médicas Básicas-Fisiología, Facultad de Medicina, Universidad de La Laguna, 38200 Tenerife, Spain;; ^b^Instituto de Tecnologías Biomédicas, Universidad de La Laguna, 38200 Tenerife, Spain

**Keywords:** large-conductance calcium- and voltage-activated potassium channels, functional coupling, ion channel macromolecular complexes, synaptic plasticity

## Abstract

Postsynaptic *N*-methyl-D-aspartate receptors (NMDARs) are crucial mediators of synaptic plasticity due to their ability to act as coincidence detectors of presynaptic and postsynaptic neuronal activity. However, NMDARs exist within the molecular context of a variety of postsynaptic signaling proteins, which can fine-tune their function. Here, we describe a form of NMDAR suppression by large-conductance Ca^2+^- and voltage-gated K^+^ (BK) channels in the basal dendrites of a subset of barrel cortex layer 5 pyramidal neurons. We show that NMDAR activation increases intracellular Ca^2+^ in the vicinity of BK channels, thus activating K^+^ efflux and strong negative feedback inhibition. We further show that neurons exhibiting such NMDAR–BK coupling serve as high-pass filters for incoming synaptic inputs, precluding the induction of spike timing–dependent plasticity. Together, these data suggest that NMDAR-localized BK channels regulate synaptic integration and provide input-specific synaptic diversity to a thalamocortical circuit.

## Significance

*N*-methyl-D-aspartate (NMDA) receptors are critical triggers for neuronal plasticity. We show that large-conductance Ca^2+^- and voltage-gated K^+^ (BK) channels serve as feedback regulators of NMDA receptor–mediated calcium influx to shape NMDA receptor–mediated synaptic potentials and consequently elevate the threshold for triggering plasticity at a subset of synapses.

Glutamate is the primary excitatory chemical transmitter in the mammalian central nervous system (CNS), where it is essential for neuronal viability, network function, and behavioral responses (1). Glutamate activates a variety of pre- and postsynaptic receptors, including ionotropic receptors (iGluRs) that form ligand-gated cation-permeable ion channels. The iGluR superfamily includes α-amino-3-hydroxy-5-methyl-4-isoxazolepropionic acid receptors (AMPARs), kainate receptors, and *N*-methyl-D-aspartate receptors (NMDARs), all of which form tetrameric assemblies that are expressed throughout the CNS (2).

NMDARs exhibit high sensitivity to glutamate (apparent half maximal effective concentration in the micromolar range) and a voltage-dependent block by Mg^2+^ (3, 4), slow gating kinetics (5), and high permeability to Ca^2+^ (6, 7) (for a review, see ref. 8). Together, these characteristics confer postsynaptic NMDARs with the ability to detect and decode coincidental activity of pre- and postsynaptic neurons: presynaptic glutamate release brings about the occupation of the agonist-binding site and AMPAR-driven postsynaptic depolarization, removing the voltage-dependent Mg^2+^ block. The coincidence of these two events leads to NMDAR activation and a Ca^2+^ influx through the channel (8, 9), which initiates several forms of synaptic plasticity (10, 11).

Large-conductance Ca^2+^- and voltage-gated K^+^ (BK) channels are opened by a combination of membrane depolarization and relatively high levels of intracellular Ca^2+^ (12, 13). In CNS neurons, such micromolar Ca^2+^ increases are usually restricted to the immediate vicinity of Ca^2+^ sources, including voltage-gated Ca^2+^ channels (VGCCs) (14–16) and ryanodine receptors (RyRs) (17, 18). In addition, Ca^2+^ influx through nonselective cation-permeable channels, including NMDARs, has also been shown to activate BK channels in granule cells from the olfactory bulb and dentate gyrus (19–21). In these neurons, Ca^2+^ entry through NMDARs opens BK channels in somatic and perisomatic regions, causing the repolarization of the surrounding plasma membrane and subsequent closure of NMDARs. Because BK channel activation blunts NMDAR-mediated excitatory responses, it provides a negative feedback mechanism that modulates the excitability of these neurons (19, 20). Thus, the same characteristics that make NMDARs key components in excitatory synaptic transmission and plasticity can paradoxically give rise to an inhibitory response when NMDARs are located in the proximity of BK channels. However, it is unclear whether functional NMDAR–BK coupling is relevant at dendrites and dendritic spines.

The barrel field area in the primary somatosensory cortex, also known as the barrel cortex (BC), processes information from peripheral sensory receptors for onward transmission to cortical and subcortical brain regions (22, 23). Sensory information is received in the BC from different nuclei of the thalamus. Among these nuclei, the ventral posterior medial nucleus, ventrobasal nucleus, and posterior medial nucleus are known to directly innervate layer 5 pyramidal neurons (BC-L5PNs) (24–27). In basal dendrites of BC-L5PN, the coactivation of neighboring dendritic inputs can initiate NMDAR-mediated dendritically restricted spikes characterized by large Ca^2+^ transients and long-lasting depolarizations (28–30), providing the appropriate environment for BK activation.

To determine whether functional NMDAR–BK coupling plays a role in synaptic transmission, and potentially synaptic plasticity, we investigated the thalamocortical synapses at basal dendrites of BC-L5PNs. We found that the suppression of NMDAR activity by BK channels occurs in the basal dendrites of about 40% of BC-L5PNs, where NMDAR activation triggers strong negative feedback inhibition by delivering Ca^2+^ to nearby BK channels. This inhibition regulates the amplitude of postsynaptic responses and increases the threshold for the induction of synaptic plasticity. Our findings thus unveil a calibration mechanism that can decode the amount and frequency of afferent synaptic inputs by selectively attenuating synaptic plasticity and providing input-specific synaptic diversity to a thalamocortical circuit.

## Results

### NMDAR Activation Opens BK Channels in BC-L5PN Basal Dendrites.

**Fig. 1.**
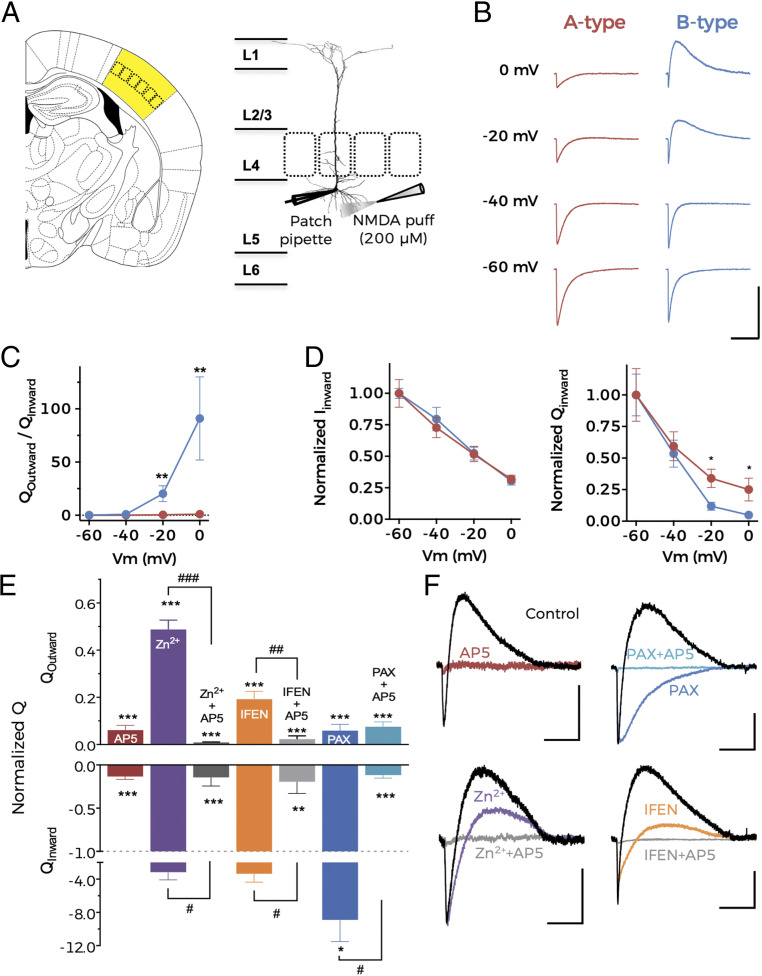
NMDAR activation opens BK channels in BC-L5PN basal dendrites. (*A*, *Left*) Schematic representation of a mouse brain slice with the BC area highlighted in yellow. (*Right*) Schematic representation of the barrel cortex depicting the BC-L5PNs under investigation. (*B*) Representative current traces obtained at the indicated holding potentials after application of NMDA to the basal dendrites. (Scale bars: 10 s and 200 pA.) (*C*) Average charge–voltage (Q–V) relationships for A-type (red) and B-type (blue) neurons. (*D*) Normalized current–voltage (*Left*) and Q–V (*Right*) relationships for NMDAR inward currents from A-type and B-type neurons. Data points in *C* and *D* represent mean ± SEM. A-type: *n* = 15, B-type: *n* = 8; *P < 0.05 and **P < 0.01 (B-type versus A-type). (*E*) Pharmacological characterization of NMDA-activated current in BC-L5PN basal dendrites. Normalized charge for the outward (Q_Outward_, *Top*) and inward (Q_Inward_, *Bottom*) component after the addition of different drugs. Data correspond to mean ± SEM, **P < 0.01 and ***P < 0.001 (treatment versus ACSF); ^#^*P* < 0.05, ^##^*P* < 0.01, and ^###^*P* < 0.01 (drug + AP5 versus drug alone). (*F*) Representative current traces obtained at −20 mV after NMDA application to the basal dendrites of B-type neurons in control conditions (ACSF, black traces) and after the application of different drugs. Traces are color-coded corresponding to the different treatments shown in *E*. (Scale bars: 5 s and 50 pA.) In *E* and *F*, AP5, D-AP5 (100 µM; *n* = 6); IFEN (5 µM; *n* = 5); PAX, paxilline (1 µM; *n* = 5); and Zn^2+^, ZnCl_2_ (100 nM; *n* = 5).

To investigate whether NMDA receptors are functionally coupled to other channel types in the basal dendrites of BC-L5PNs, we obtained whole-cell voltage-clamp recordings from pyramidal neurons located beneath layer 4 barrels in acute mouse brain slices (*n* = 108) (Fig. 1*A*). Localization and large pyramidal-shaped somata suggest that we predominantly recorded from layer 5a neurons. In the presence of Mg^2+^-free artificial cerebrospinal fluid (ACSF) supplemented with tetrodotoxin (TTX; 1 µM) and glycine (10 µM), puff application of 200 µM NMDA to the basal dendrites of BC-L5PNs evoked a NMDAR-resembling inward current in all neurons (Fig. 1*B*). Remarkably, about 40% of the BC-L5PNs developed a slower outward current at holding potentials more positive than −40 mV (Fig. 1*B*), which showed a clear dependence on membrane voltage (Fig. 1*C*). These two populations of BC-L5PNs were assigned as A-type neurons (lacking the outward current; *n* = 65, 60.2%) and B-type neurons (showing the outward current; *n* = 43, 39.8%). Inward current amplitude was directly proportional to the holding potential in both populations (Fig. 1 *D*, *Left*), indicating that a similar molecular species carries the inward current in both neuronal types. Interestingly, the activation of the outward current in B-type neurons significantly reduced net inward current flow, decreasing the inward charge transfer (Fig. 1 *D*, *Right*). When NMDA was applied at the same distance from the soma as it was previously, but toward layer 4, only the inward current was observed (*SI Appendix*, Fig. S1). These results suggest that NMDA can evoke an outward current in the basal dendrites of B-type BC-L5PNs but not in the soma, oblique/truncated dendrites, or the initial segment of the apical dendrite. Our findings are in contrast to what has been found in other neurons such as hippocampal CA1 pyramidal cells, where NMDA-dependent outward currents were observed at the soma (21) but not at dendrites (*SI Appendix*, Fig. S2).

We performed the pharmacological characterization of inward and outward currents in B-type neurons at a holding potential of −20 mV (Fig. 1*E*). The complete abrogation of inward current by the selective NMDAR antagonist D-AP5 (AP5; 100 µM) confirmed that it was due to the cation flow through NMDARs. Moreover, because the outward current was also abolished in the presence of AP5, we surmised that NMDAR activation is mandatory for outward current generation (Fig. 1 *E* and *F*). Selective inhibition of GluN2A- or GluN2B-containing NMDARs using 100 nM ZnCl_2_ or 5 µM ifenprodil (IFEN), respectively, partially suppressed the outward current (Fig. 1 *E* and *F*). This suggests that an inward current flow through both GluN2A- and GluN2B-containing NMDARs leads to an activation of the outward current in B-type neurons.

The voltage dependence and direction of net ionic flow (Fig. 1*C*) suggests that the NMDAR-dependent outward current is driven by a voltage-dependent K^+^ channel. Because this current is reminiscent of that carried by BK channels in granule cells from the olfactory bulb and dentate gyrus (19, 20), we applied NMDA to basal dendrites of BC-L5PNs in the presence of the specific BK channel pore blocker paxilline (1 µM) (31). Paxilline completely abolished the NMDAR-dependent outward current without eliminating the inward component, suggesting that NMDAR activation in the basal dendrites of B-type neurons causes BK channels to open and therefore that NMDARs and BK channels are functionally coupled.

### NMDARs and BK Channels Are Within Functional Proximity in B-type BC-L5PNs.

Our results suggested that activation of NMDARs in the basal dendrites of B-type BC-L5PNs provides the Ca^2+^ needed to activate BK channels. To confirm this hypothesis, we recorded an NMDA-evoked current in the presence of the intracellular Ca^2+^ chelators 1,2-bis(*o*-aminophenoxy)ethane-*N*,*N*,*N′*,*N′*-tetraacetic acid (BAPTA) and ethylene glycol-bis(β-aminoethyl ether)-*N*,*N*,*N′*,*N′*-tetraacetic acid (EGTA), which we delivered through the recording pipette. BAPTA and EGTA are useful biochemical tools to estimate the linear distance that Ca^2+^ diffuses from its source (32). This approach has been previously used to calculate the distance between channels in BK-VGCC macrocomplexes (e.g., refs. 33 and 34 but see also ref. 14 and the references within). Both chelators present similar affinities for Ca^2+^, but BAPTA has a faster association rate than EGTA. Therefore, an outward current through BK channels would be recorded in the presence of EGTA, but not BAPTA, if both channels are located in close proximity. If the distance between both channels is larger, Ca^2+^ entering through NMDARs would be captured by both chelators before reaching BK, and no outward current would be observed. Taking into account the dendritic complexity of pyramidal neurons, we used higher concentrations of chelators than those described in previous studies assessing NMDAR–BK somatic associations in hippocampal neurons (20).

**Fig. 2.**
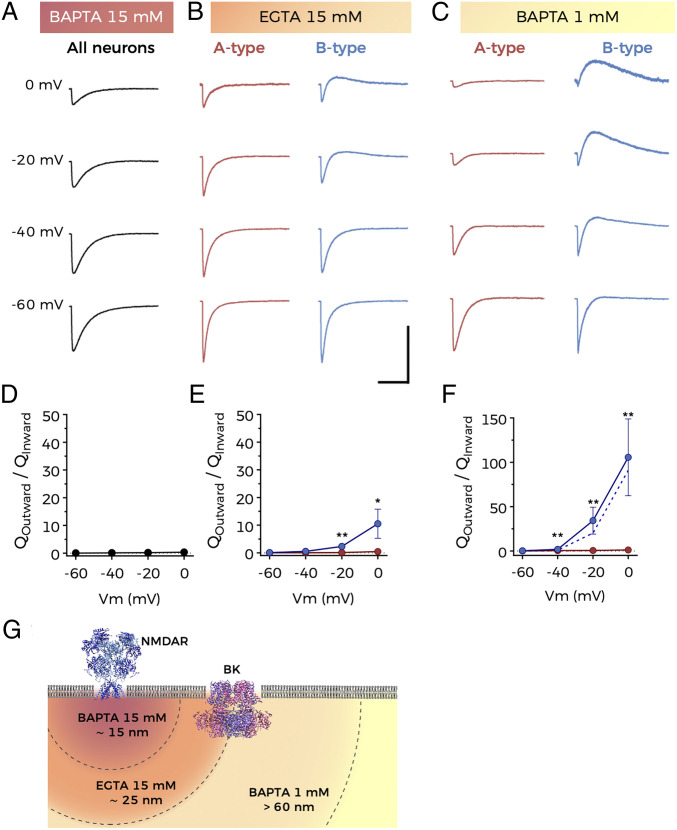
NMDARs and BK channels are within functional proximity in B-type BC-L5PNs. (*A–C*) Representative current traces obtained at the indicated holding potentials after application of NMDA to the basal dendrites of BC-L5PNs in the presence of (*A*) 15 mM BAPTA, (*B*) 15 mM EGTA, and (*C*) 1 mM BAPTA in the recording pipette. (Scale bars: 10 s and 200 pA.) (*D–F*) Average charge–voltage relationships corresponding to experiments described in *A–C*; Data points represent mean ± SEM; (*D*) *n* = 18, (*E*) A-type: *n* = 8, B-type: *n* = 5, (*F*) A-type: *n* = 9, B-type: *n* = 5; blue dashed line in *F* represents data taken from Fig. 1*C* (B-type neurons) for comparison; *P < 0.05 and **P < 0.01 (B-type versus A-type). (*G*) Schematic representation of the relative location of NMDARs and BK channels in the plasma membrane. Distances are estimated from the experimental data.

We failed to observe NMDA-induced outward currents in any BC-L5PNs in the presence of 15 mM BAPTA (Fig. 2 *A* and *D*), confirming that Ca^2+^ entry through NMDARs is responsible for the activation of BK channels. However, when the slower chelator EGTA was used at the same concentration, two populations of BC-L5PNs could be distinguished (Fig. 2*B*). As in control conditions, the outward current in B-type neurons activated at holding potentials positive to −40 mV and exhibited a clear dependence on membrane voltage, but its amplitude was significantly reduced due to substantial Ca^2+^ chelation (compare Fig. 2*E* with 1*C*). When the BAPTA concentration was reduced to 1 mM, the outward current could again be recorded (Fig. 2*C*) and was quantitatively identical to that obtained in control conditions (compare Fig. 2*F* with Fig. 1*C*). Using described Ca^2+^ linear diffusion data in the presence of BAPTA and EGTA (32), we estimated that NMDAR and BK channels are located within 15 to 60 nm of each other in the basal dendrites of B-type BC-L5PNs (Fig. 2*G*); in close enough proximity to explain the specific functional coupling that we observed.

### Both GluN2A- and GluN2B-Containing NMDARs Can Functionally Couple to BK Channels.

**Fig. 3.**
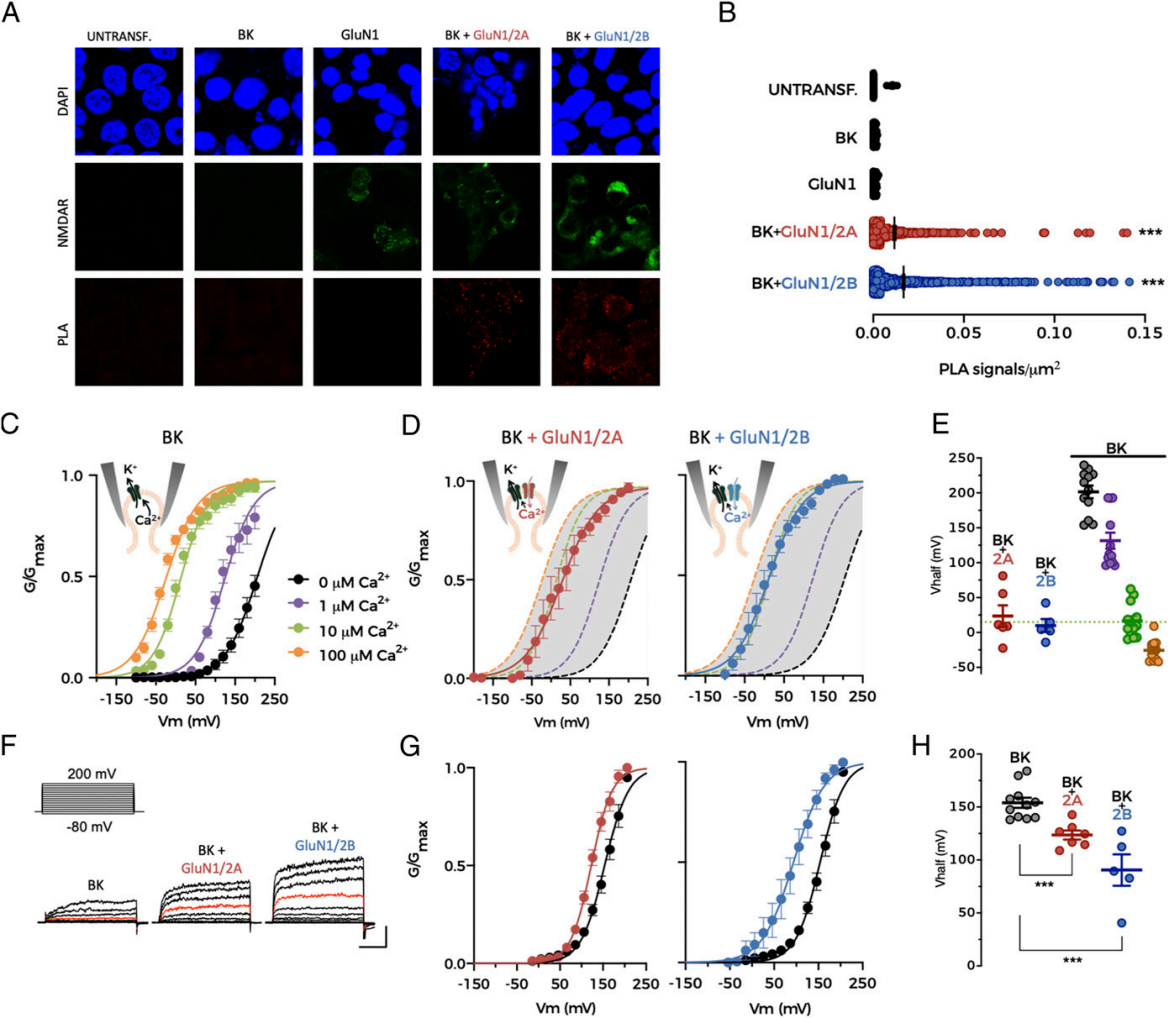
Both GluN2A- and GluN2B-containing NMDARs can functionally couple to BK channels. (*A*) Representative confocal microscopy images of PLA experiments in HEK293 cells expressing the protein combinations indicated at the top of the images. Each row corresponds to an imaging channel (*Top*, DAPI, blue; *Middle*, NMDAR, green; *Bottom*, PLA, red). (*B*) Average PLA signals/µm^2^ for all conditions shown in *A*. Data points represent individual measurements. Four independent experiments were performed for each condition. Black lines represent mean ± SEM, ***P < 0.001 versus untransfected cells (UNTRANSF), BK alone (BK), and GluN1 alone (GluN1). (*C*) Normalized G–V relationships obtained from excised inside-out patches expressing BK channels alone (*n* = 12) in the presence of different intracellular Ca^2+^ concentrations in symmetrical K^+^ solutions. Data points represent mean ± SEM. Lines represent the best fit of a Boltzmann equation to the data. Ca^2+^ concentrations are color-coded as indicated in the legend. (*D*) Normalized G–V relationships from excised inside-out patches co-expressing BK channels with GluN1/GluN2A (*Left*; *n* = 6) or GluN1/GluN2B (*Right*; *n* = 5) in the absence of intracellular Ca^2+^, with 200 µM NMDA/10 µM glycine in the pipette. Data points represent mean ± SEM. Lines represent the best fit of a Boltzmann equation to the data. (*E*) V_half_ values obtained from experiments depicted in *C* and *D*. Data points represent individual measurements, and lines represent mean ± SEM. (*F*) Representative current traces recorded in physiological Na^+^ solutions from excised inside-out patches containing BK alone (*Left*), BK+GluN1/GluN2A (*Middle*), and BK+GluN1/GluN2B (*Right*). (Scale bars: 30 ms and 2,000 pA.) Currents recorded at +140 mV are highlighted in red. (*G*) Normalized G–V relationships from excised inside-out patches containing BK and GluN1/GluN2A (*Left*, red; *n* = 6) or GluN1/GluN2B (*Right*, blue; *n* = 5) in solutions containing physiological concentrations of Na^+^ (see *Methods*). Black traces in both graphs correspond to G–V curves for BK channels expressed alone (*n* = 11). Data points represent mean ± SEM. Lines represent the best fit of a Boltzmann equation to the data. (*H*) Summary of V_half_ values from experiments in *G*. Data points represent individual measurements and lines represent mean ± SEM, ***P < 0.001 versus BK alone.

Because GluN2 subunits determine the deactivation kinetics and ion conductance of NMDARs (35), we reasoned that the molecular composition of NMDARs might affect NMDAR–BK coupling efficiency. We first tested whether BK preferentially associates with specific NMDAR subunit combinations using a proximity ligation assay (PLA). In agreement with our pharmacological data (Fig. 1*F*), positive PLA signals were observed for human embryonic kidney (HEK293T) cells co-expressing BK and either GluN1/GluN2A or GluN1/GluN2B NMDARs (Fig. 3*A*), without any preference between GluN2A and GluN2B subunits (Fig. 3*B*).

Having confirmed that NMDARs and BK channels are located in close proximity in the plasma membrane when co-expressed in HEK293T cells, we characterized functional coupling between specific channel/subunit combinations. By using the inside-out configuration of the patch-clamp technique, we were able to monitor channel function while controlling the “intracellular” Ca^2+^ concentration in the bath solution (36). NMDARs were activated by including 200 µM NMDA and 10 µM glycine in the “extracellular” pipette solution. Under these conditions, the desensitization of NMDAR is significantly reduced (37). The relative conductance (G) of BK channels exposed to different intracellular Ca^2+^ concentrations (from 0 to 100 µM) in patches from cells expressing BK channels but no NMDARs (Fig. 3*C*) corresponded to typical Ca^2+^-dependent activation curves for these channels (38, 39). We reasoned that the addition of Ca^2+^ to the intracellular side of the patch through a Ca^2+^ source such as NMDARs would result in a leftward shift of the BK activation curve in a zero Ca^2+^ bath solution. This would be favored if the channels were closely located, allowing Ca^2+^ to activate BK before being diluted in the large Ca^2+^-free bath solution (following an analogous reasoning than the experiments using chelators above). Indeed, the co-expression of BK channels with either GluN1/GluN2A or GluN1/GluN2B NMDARs produced a significant leftward shift of the BK activation curve (Fig. 3*D*). Interestingly, the shift resulted in an activation curve comparable to that recorded with 10 µM intracellular Ca^2+^ in patches expressing BK alone (Fig. 3*E* and *SI Appendix*, Table S1). Of note, the reduction of the driving force for Ca^2+^ at very positive voltages was not paralleled by diminished values in the conductance–voltage (G–V) curves as is commonly observed in VGCC–BK coupling experiments (15), probably due to increased permeation of K^+^ through NMDAR (40). These results suggest that NMDAR activation increases Ca^2+^ concentration in the close vicinity of BK channels, favoring their activation.

NMDARs are nonselective cation channels that are permeable to Na^+^, K^+^, and Ca^2+^ (2, 8). Under physiological conditions, the proportion of NMDAR current carried by Ca^2+^ corresponds to 10 to 15% of the total current (41–43). However, because the abovementioned inside-out patch experiments were performed in the absence of Na^+^, the inward current through NMDARs could only be due to Ca^2+^ ions, whose permeability is increased as extracellular Na^+^ concentration is reduced (7). We may therefore be overestimating the effects of NMDAR-dependent Ca^2+^ activation on BK channels in our zero Na^+^ experimental conditions. Nevertheless, activation of GluN1/GluN2A or GluN1/GluN2B NMDARs in excised inside-out patches using physiological concentrations of Na^+^ still produced a leftward shift in the BK activation curve (Fig. 3 *F*–*H*). Interestingly, GluN1/GluN2B produced larger shifts than GluN1/GluN2A.

Taken together, these results demonstrate that both GluN2A- and GluN2B-containing NMDARs can provide sufficient Ca^2+^ for BK activation, consistent with our data from BC-L5PN basal dendrites (Fig. 1*F*).

### A Subpopulation of Regular-Spiking BC-L5PNs Exhibit NMDAR–BK Functional Coupling.

**Fig. 4.**
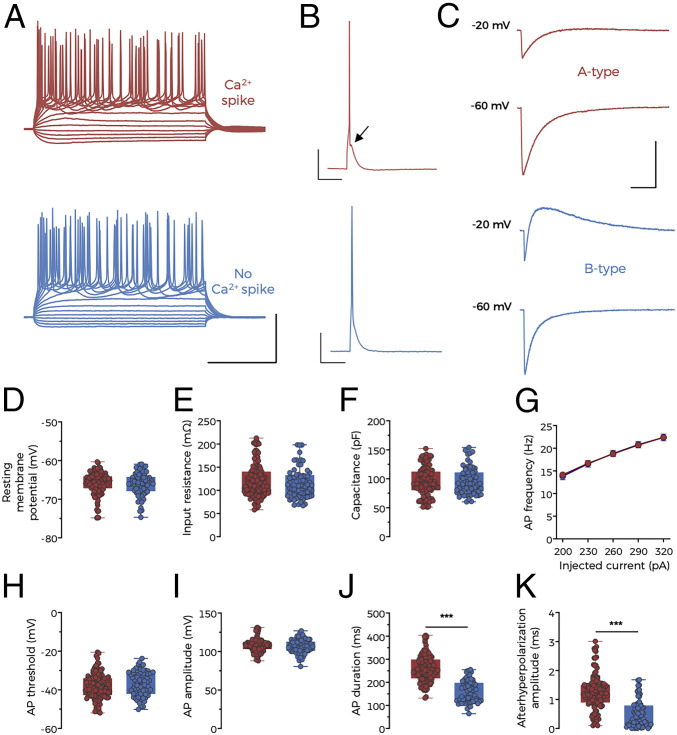
A subpopulation of regular-spiking BC-L5PNs exhibit NMDAR–BK functional coupling. (*A*) Representative current-clamp traces elicited with 500-ms depolarizing steps from −100 to 320 pA in A-type (red) and B-type (blue) BC-L5PNs. (Scale bars: 200 ms and 50 mV.) (*B*) Single action potentials recorded from the same neurons shown in *A*. The arrow points to the Ca^2+^ spike apparent in A-type BC-L5PNs. (Scale bars: 100 ms and 20 mV.) (*C*) NMDA-evoked currents recorded from the same neurons depicted in *A* and *B* at holding potentials of −20 and −60 mV with a Mg^2+^-free external solution containing 1 µM TTX and 10 µM glycine. (Scale bars: 5 s and 150 pA.) (*D–K*) Values for resting membrane potential (*D*), input resistance (*E*), cell capacitance (*F*), action potential frequency (*G*), action potential threshold (*H*), action potential amplitude (*I*), action potential duration (*J*), and afterhyperpolarization amplitude (*K*). Data correspond to individual measurements (symbols) and median and 25th to 75th percentiles (boxes) from A-type (red, *n* = 130) and B-type (blue, *n* = 67) BC-L5PNs, except for *G*, where data points represent mean ± SEM. In *J* and *K*, ***P < 0.001 (B-type versus A-type).

BC neurons can be classified into fast-spiking nonpyramidal GABAergic interneurons and regular-spiking or intrinsically bursting pyramidal neurons according to their electrophysiological properties (24, 44, 45). Regular-spiking neurons from layers 5 and 6, where regularly timed trains of action potentials are observed in response to somatic current injection, can be further characterized by the presence or absence of a depolarizing afterpotential known as a Ca^2+^ spike (24, 46). To determine whether NMDAR–BK functional coupling is restricted to a specific neuronal subtype, we performed whole-cell current-clamp recordings in regular-spiking BC-L5PNs in mouse brain slices (*n* = 197) in the presence of physiological concentrations of Mg^2+^ and Ca^2+^. By inducing single action potentials in BC-L5PNs, we confirmed the presence of two populations of neurons that either exhibited a Ca^2+^ spike (*n* = 130, 66.0%) or not (*n* = 67, 34.0%) (Fig. 4 *A* and *B*), similar to those previously described (24, 46, 47).

We next investigated whether these two populations of BC-L5PNs could be correlated with NMDAR–BK functional coupling by subsequently perfusing the brain slices with a Mg^2+^-free solution supplemented with TTX (1 µM) and glycine (10 µM). Voltage-clamp recordings were carried out in the same neurons upon a puff application of NMDA to their basal dendrites (Fig. 4*C*). None of the BC-L5PNs exhibiting a Ca^2+^ spike presented an outward current in response to NMDAR activation. Conversely, all BC-L5PNs lacking a Ca^2+^ spike showed a robust BK outward current after NMDA application (Fig. 4 *B* and *C*). No further intrinsic or evoked differences were observed between A-type and B-type neurons (Fig. 4 *D*–*I*), except for those that were directly related to the presence of the Ca^2+^ spike (Fig. 4 *J* and *K*). We therefore conclude that the presence of NMDAR–BK coupling in basal dendrites is restricted to a population of regular-spiking BC-L5PNs that are characterized by the absence of Ca^2+^ spikes.

### BK-Dependent Inhibition of NMDARs Reduces Amplitude and Slows Down Kinetics of the Postsynaptic Response.

Having demonstrated that BK channels in the basal dendrites of BC-L5PNs are activated after Ca^2+^ entry through proximal NMDARs, we predicted that this coupling would give rise to a negative feedback loop similar to that described for the coupling of VGCCs and BK channels in presynaptic terminals (48) or NMDARs and small-conductance Ca^2+^-activated K^+^ channels (SK) in postsynaptic terminals (49). In such a scheme, NMDAR activation and the subsequent entry of Ca^2+^ would open BK channels, which would repolarize the membrane due to an outward flux of K^+^ and therefore reinstate a voltage-dependent Mg^2+^ block of NMDARs, in turn truncating Ca^2+^ entry. If that were the case, the presence of postsynaptic NMDAR–BK coupling in B-type neurons should have a significant impact on synaptic transmission. Indeed, our finding that BK activation reduced the NMDAR-mediated inward current in B-type neurons (Fig. 1*D*) suggested the presence of such a negative feedback loop.

**Fig. 5.**
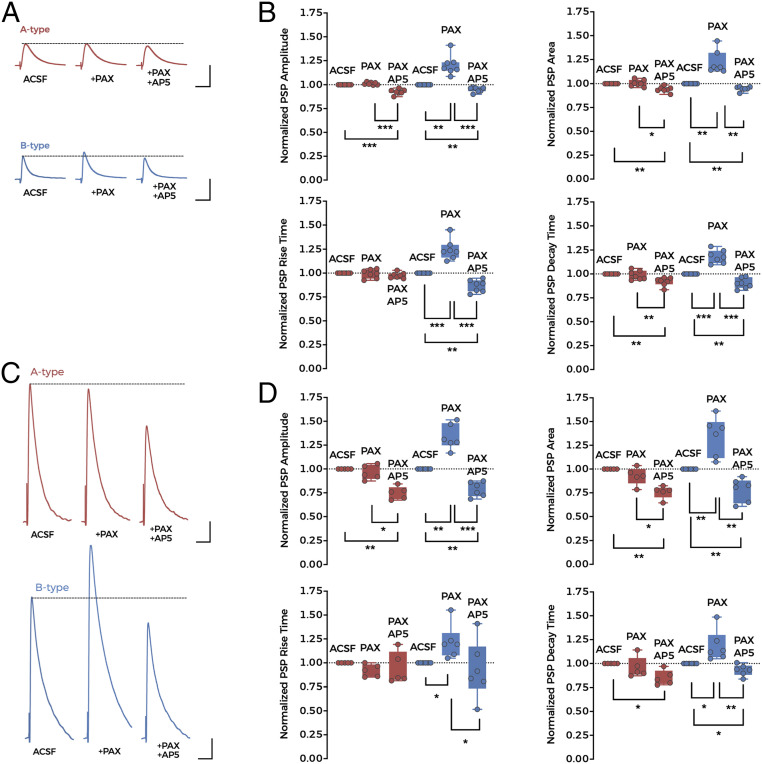
BK-dependent inhibition of NMDARs reduces postsynaptic response amplitude. (*A*) Representative synaptically evoked PSP traces recorded from A-type (*Top*, red) and B-type (*Bottom*, blue) neurons in control conditions (ACSF, *Left*) after the application of 1 µM paxilline (PAX, *Middle*) and with 1 µM PAX plus 100 µM AP5 (PAX + AP5, *Right*). (Scale bars: 50 ms and 3 mV.) (*B*) Values for the normalized PSP amplitude (*Top Left*), area (*Top Right*), rise time (*Bottom Left*), and decay time (*Bottom Right*) in the experimental conditions in *A*. Data points represent individual measurements (*n* = 7 in both types of neuron), and boxes represent median and 25th to 75th percentile values. (*C*) Representative synaptically evoked PSP traces recorded from A-type (*Top*, red) and B-type (*Bottom*, blue) neurons after increasing the electrical stimulation intensity applied to the afferent inputs. Conditions were the same as in *A* (ACSF, PAX, PAX + AP5). The pipette solution included 2 mM QX-314 to avoid action potential firing. (Scale bars: 50 ms and 3 mV.) (*D*) Values for PSP amplitude (*Top Left*), area (*Top Right*), rise time (*Bottom Left*), and decay time (*Bottom Right*) for the experimental conditions shown in *C*. Data points represent individual measurements, and boxes represent median and 25th to 75th percentile values. A-type: *n* = 5; B-type: *n* = 6. In *B* and *D*, *P < 0.05, **P < 0.01, and ***P < 0.001.

To investigate the effect of NMDAR–BK coupling on synaptic transmission, we electrically stimulated the afferent inputs to BC-L5PN basal dendrites and recorded evoked postsynaptic potentials (PSPs) in physiological conditions (with Mg^2+^ in the external solution). Presynaptic stimulation of basal afferent inputs was performed at the limit between layers 5 and 6 to activate ascending thalamocortical fibers (50, 51), many of which are known to make direct contacts with BC-L5PNs (24–27). The presence or absence of a Ca^2+^ spike in evoked action potentials allowed us to classify BC-L5PNs as either A-type or B-type. Stimulation intensity was then adjusted to obtain 3 to 5 mV PSPs in all recorded neurons (Fig. 5*A*). These values correspond to depolarizations of >30 mV occurring at basal dendrites, taking into account the signal attenuation from dendrites to the soma (28, 29). A BK channel block using a bath perfusion of 1 µM paxilline increased PSP amplitude and slowed PSP kinetics (Fig. 5*B*) (but only in B-type neurons), suggesting an increase of postsynaptic NMDAR availability in B-type neurons. Although a selective inhibition of BK channels at presynaptic sites that target B-type neurons cannot be excluded, the lack of any effect of paxilline on PSPs in A-type neurons, characterized by the absence of NMDAR–BK functional complexes, supports the absence of effects of presynaptic BK channels in the synapses we studied. The further addition of AP5 (100 µM) to the perfusate accelerated PSP kinetics and completely abolished the PSP amplitude increase below control levels (Fig. 5*B*), revealing that the AMPAR component is not increased in our experimental conditions. In summary, this data suggests that NMDAR-dependent activation of postsynaptic BK channels reduces the contribution of NMDARs to PSPs, thereby regulating synaptic transmission.

The coactivation of clustered neighboring basal inputs to BC-L5PNs initiates local dendritic NMDAR-dependent spikes that are characterized by large Ca^2+^ transients (28, 30). We induced NMDA-dependent Ca^2+^ spikes by increasing the stimulation intensity applied to the afferent inputs to BC-L5PN basal dendrites while blocking action potentials by including the Na^+^ channel blocker QX-314 (2 mM) in the recording pipette. As expected, larger PSPs were observed in these experimental conditions (Fig. 5*C*). Paxilline (1 µM) induced a further increase in PSP amplitude in B-type neurons but had no effect on A-type neurons (Fig. 5 *C* and *D*). The further addition of AP5 (100 µM) reversed the PSP amplitude increase in B-type neurons to below control values and to a similar amplitude in both neuronal types (Fig. 5 *C* and *D*). It must be noted that in our experimental conditions, we may be underestimating this effect, taking into account that QX-314 may partially inhibit BK channels, similarly to other quaternary ammonium compounds. These results demonstrate that BK channels in the basal dendrites of B-type neurons are able to abrogate the NMDAR current, including under conditions in which NMDAR spikes are taking place.

### NMDAR–BK Coupling Increases the Threshold for Induction of Synaptic Plasticity.

The inhibitory effect of NMDAR**–**BK coupling on synaptic transmission in BC-L5PNs led us to wonder whether it may play a role in other physiological mechanisms, including forms of long-term synaptic plasticity involving NMDAR activation (10, 11). Spike timing–dependent plasticity (STDP) is one such mechanism and relies on the precise coincidence of presynaptic and postsynaptic activity (11, 52). The timing and order of presynaptic and postsynaptic action potentials determine the direction of the change in synaptic strength: a presynaptic action potential followed by a postsynaptic action potential within a window of tens of milliseconds results in long-term potentiation (LTP), whereas the reverse order within a similar timeframe results in long-term depression (LTD) (11, 52, 53). Both mechanisms are dependent on NMDAR activation (54), but only spike timing–dependent LTP (t-LTP) depends on postsynaptic NMDAR activation and the consequential rise in dendritic spine Ca^2+^ concentration (54) (*SI Appendix*, Fig. S3). Therefore, this experimental approach allowed us to restrict our study to the postsynaptic mechanisms related to NMDAR–BK functional coupling. Because NMDAR activity is blunted by BK activation in the basal dendrites of B-type BC-L5PNs, we hypothesized that t-LTP would be less prominent in these neurons compared to A-type BC-L5PNs, or possibly absent. We therefore studied the effects of pairing pre- and postsynaptic action potentials in A-type and B-type BC-L5PNs.

**Fig. 6.**
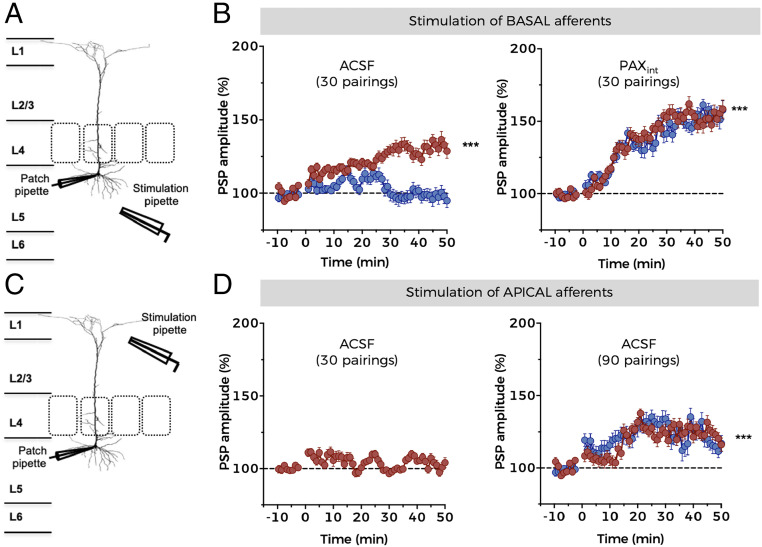
NMDAR–BK coupling increases the threshold for induction of synaptic plasticity. (*A*) Schematic representation of the experimental design used to induce t-LTP via stimulation of basal afferent inputs to BC-L5PNs. (*B*) Development of t-LTP over time in A-type (red) and B-type (blue) neurons in control conditions (ACSF, *Left*) and in the presence of 500 nM intracellular paxilline (PAX, *Right*). Data points represent mean ± SEM. A-type (ACSF): *n* = 6; B-type (ACSF): *n* = 4; A-type (PAX): *n* = 6; B-type (PAX): *n* = 4. ***P < 0.001 (t-LTP versus basal conditions). (*C*) Schematic representation of the experimental design used to induce t-LTP via stimulation of apical afferent inputs to BC-L5PNs. (*D*) Development of t-LTP over time in A-type (red) and B-type (blue) neurons in control conditions (ACSF) using 30 (*Left*) or 90 (*Right*) STDP pairings. Data points represent mean ± SEM. A-type (30 pairings): *n* = 3; A-type (90 pairings): *n* = 6; B-type (90 pairings): *n* = 3.

We measured PSPs evoked by electrical stimulation of basal afferent inputs to BC-L5PNs before and after pre–post pairings at 0.20 Hz (Fig. 6*A*). A low number of pre–post associations (30 pairings) induced t-LTP in A-type but not B-type neurons (Fig. 6 *B*, *Left*). This is consistent with a reduction in the amount of Ca^2+^ entering the basal dendrites of B-type neurons due to NMDAR-dependent activation of BK channels. The difference between the two populations was abolished when 500 nM paxilline was included in the recording pipette (Fig. 6 *B*, *Right*), suggesting that the release of NMDARs from BK-induced negative feedback favors t-LTP by reducing the threshold for its induction. This action of paxilline applied to the postsynaptic cell is enough to abolish the BK channel effect on plasticity. Although paxilline crosses cell membranes and could therefore conceivably affect BK channels beyond the patched cell, both dilution by the larger extracellular volume fraction and bath perfusion and the reduced efficiency of inhibition from the external side of the membrane (31) would all serve to reduce any paxilline reaching neighboring cells to negligible levels. The degree of t-LTP in both neuronal types in the presence of paxilline was significantly greater than that elicited in A-type neurons in control conditions (Fig. 6 *B*, *Right* versus *Left*), consistent with the key role of BK channels in the regulation of neuronal excitability (12). Interestingly, these data imply that BK channels are functionally expressed in both A-type and B-type BC-L5PNs, which we confirmed by recording BK currents in both types of BC-L5PN (*SI Appendix*, Fig. S4).

As our data indicated that NMDAR–BK coupling is restricted to the basal dendrites of BC-L5PNs, we asked whether the difference in the synaptic plasticity threshold between A-type and B-type neurons was limited to basal dendrites or could also occur in apical dendrites. We electrically stimulated the afferent inputs to the apical dendrites of BC-L5PNs (Fig. 6*C*) and carried out 30 pre–post pairings at 0.20 Hz. This failed to induce synaptic plasticity in any of the neurons tested (Fig. 6 *D*, *Left*), but when the number of pre–post pairings was increased to 90, t-LTP was induced in both types of neurons (Fig. 6 *D*, *Right*). The potentiation was of a similar amplitude in both A- and B-type neurons but significantly lower than that induced in basal dendrites by 30 pairings (compare Fig. 6 *D*, *Right* with Fig. 6 *B*, *Left*). These results confirm that functional NMDAR–BK coupling occurs exclusively in the basal dendrites of B-type BC-L5PNs, where it increases the threshold for synaptic plasticity and therefore modulates neuronal circuits involving these dendrites.

### A High Number and Frequency of Pre–Post Pairings Relieves BK-Dependent NMDAR Inhibition.

**Fig. 7.**
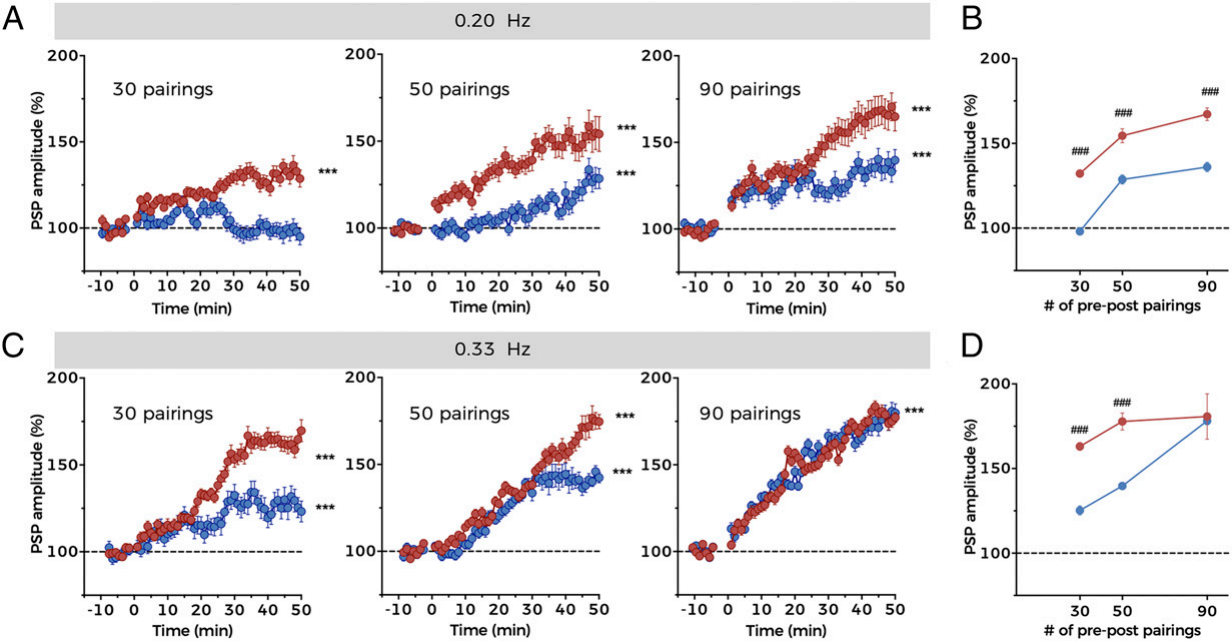
A high number and frequency of pre–post pairings relieves BK-dependent NMDAR inhibition. (*A*) Time course of t-LTP development over time in A-type (red) and B-type (blue) neurons using 30 (*Left*), 50 (*Middle*), or 90 (*Right*) pairing STDP protocols at frequency of 0.20 Hz using the experimental design represented in Fig. 6*A*. Data points represent mean ± SEM. A-type (30 pairings): *n* = 6, B-type (30 pairings): *n* = 4; A-type (50 pairings): *n* = 6; B-type (50 pairings): *n* = 4; A-type (90 pairings): *n* = 6; B-type (90 pairings): *n* = 5. Data in *Left* (30 pairings) are the same as in Fig. 6*B*. (*B*) Summary of t-LTP extent for A-type (red) and B-type (blue) neurons under the experimental conditions depicted in *A*. (*C*) Time course of t-LTP development over time in A-type (red) and B-type (blue) neurons using 30 (*Left*), 50 (*Middle*), or 90 (*Right*) pairing STDP protocols at a frequency of 0.33 Hz. Data points represent mean ± SEM. A-type (30 pairings): *n* = 6, B-type (30 pairings): *n* = 4; A-type (50 pairings): *n* = 7; B-type (50 pairings): *n* = 5; A-type (90 pairings): *n* = 5; B-type (90 pairings): *n* = 6. (*D*) Summary of t-LTP extent for A-type (red) and B-type (blue) neurons under the experimental conditions depicted in *C*. In *A* and *C*, ***P < 0.001 (t-LTP versus basal conditions). In *B* and *D*, ^###^*P* < 0.001 (B-type versus A-type). See also *SI Appendix*, Table S1.

Our results indicated that BK reduces, but does not completely abolish, the influx of ions through NMDARs and therefore increases the threshold for the induction of synaptic plasticity (Fig. 6*B*). We reasoned that by tuning the experimental conditions to induce greater NMDAR activation, the concentration of Ca^2+^ in postsynaptic terminals would eventually reach sufficient levels to induce plasticity in B-type BC-L5PNs. In A-type neurons, increasing the number of pre–post pairings to 50 or 90 increased the extent of t-LTP as previously described in other brain areas including the hippocampus (55–57). Importantly, we were also able to induce t-LTP in B-type neurons by increasing the number of pre–post associations (Fig. 7*A*), confirming that sufficient NMDAR activation can overcome the higher threshold for plasticity in these neurons. However, the extent of PSP potentiation in B-type neurons was significantly less than in A-type neurons for a given number of pairings (Fig. 7*B*). These results therefore demonstrate that NMDAR–BK coupling regulates the threshold to induce synaptic plasticity in the basal dendrites of B-type BC-L5PNs.

The magnitude of t-LTP can be also regulated by changing the frequency or modifying the time window of pairings (9, 54), although the latter results in considerable STDP variability depending on the synapse studied (9). We hypothesized that by increasing the frequency of pairings, a condition would be reached in which the inhibitory effect of BK on NMDARs completely disappears. For this purpose, we applied 30, 50, and 90 pairings at a frequency of 0.33 Hz (pre–post pairings delivered every 3 s) and found that the degree of t-LTP in each condition was greater than that at 0.20 Hz for both types of neurons (compare Fig. 7*C* with 7*A*). Furthermore, there was no difference in the extent of PSP potentiation between A-type and B-type neurons following the delivery of 90 pairings (Fig. 7*D*). Thus, the inhibitory effect of BK on NMDARs can be abolished with high rates of pre- and postsynaptic coincident activity. Moreover, although t-LTP appeared to saturate following 50 pairings in A-type neurons subjected to 0.33 Hz stimulation, B-type BC-L5PNs remained able to discriminate between the number of pairings in the protocol.

## Discussion

We have described the functional association of NMDARs and BK channels and the role of this coupling in synaptic function in the basal dendrites of a population of regular-spiking BC-L5PNs. NMDAR activation and the subsequent Ca^2+^ entry promotes BK opening, which repolarizes the cell membrane and halts NMDAR activity. Both GluN2A- and GluN2B-containing NMDARs can activate BK channels, although our data suggest that GluN2B-containing NMDARs are more efficient. The functional coupling of NMDARs and BK channels in the basal dendrites of this specific BC-L5PN population modulates synaptic transmission and produces an increase in the threshold for the induction of synaptic plasticity, indicating that NMDAR–BK association critically influences how specific neuronal types integrate afferent synaptic inputs. In fact, NMDAR–BK functional coupling bestows B-type BC-L5PNs with the ability to work as high-pass filters of incoming inputs, depending on the number and frequency of afferent stimuli.

In the CNS, BK channels can couple to different Ca^2+^-conducting channels, including VGCCs (14–16) and RyRs (17, 18). Such Ca^2+^ sources provide the Ca^2+^ needed for BK activation, but membrane depolarization is generally provided by a coincident action potential (48). Interestingly, NMDAR activation can provide both the membrane depolarization and the Ca^2+^ entry required for BK activation (8), particularly in restricted compartments such as the dendritic spine, where Ca^2+^ concentrations reach micromolar levels after NMDAR activation (58, 59). In contrast to previous studies using Mg^2+^-containing ACSF (19, 20), we initially observed the functional association of NMDARs and BK channels in the absence of Mg^2+^. These data revealed that NMDAR-dependent BK activation occurs at potentials positive to −40 mV within a range of potentials at which the NMDAR-dependent current is maximal (3, 4). This was subsequently corroborated in synaptic transmission experiments performed in the presence of Mg^2+^ in which the inhibition of NMDARs by BK channels became evident.

Consistent with previous studies (19, 20), our results showed that NMDAR-mediated increases in Ca^2+^ concentration are required in the immediate vicinity of BK channels in order to influence their activation. Thus, the coupling mechanism relies on the close proximity of NMDARs and BK channels in the plasma membrane. Our experiments using Ca^2+^ chelators allowed us to estimate that the two proteins must be situated within 15 to 60 nm of each other for functional coupling to occur. However, this estimate must be taken with caution, as the exact concentration of chelators that reach dendrites is not known. In fact, our experiments using PLAs suggest that the maximum distance between the two channels may be even shorter (below 40 nm). The close association of these channels in the soma has been also shown by coimmunoprecipitation and biochemical approaches (20). Our results demonstrate strong functional coupling between NMDARs and BK channels in the basal dendrites of BC-L5PNs, regardless of whether the channels physically interact with each other. Further work is needed to determine what fraction of this functional association is due to looser coupling and/or the summation from multiple NMDARs.

Our work provides evidence for a functional role of NMDAR–BK coupling in neuronal dendrites. This result differs from previous studies in which functional coupling between NMDARs and BK channels has been observed in the soma of granule cells from the olfactory bulb (19) and dentate gyrus (20). Some evidence also points to NMDAR–BK interactions in hippocampal CA1 pyramidal neuron somata (21) but not dendrites (49) (*SI Appendix*, Fig. S2). At the soma, the NMDAR-dependent activation of BK channels would likely constitute a mechanism that regulates action potential shape and controls neuronal excitability independently of dendritic input.

In dendrites, NMDAR–BK coupling would generate a negative feedback mechanism that could have dramatic effects on synaptic transmission and forms of synaptic plasticity that involve NMDARs and Ca^2+^ entry. Here, we have demonstrated that B-type BC-L5PNs exhibiting NMDAR–BK functional coupling show reduced synaptic transmission and have a higher threshold for the induction of long-term synaptic plasticity. This phenomenon of selective plasticity attenuation is restricted to the basal dendrites of these neurons, as the effect was not observed when afferent inputs to apical dendrites were stimulated. Similar basal versus apical polarity differences that affect synaptic input integration have been described in other brain areas, including the hippocampus (60).

A concern from this study might be the potential confounding factor of presynaptic BK channels, which may impact on the amount of glutamate released. Although our experiments do not specifically address the presence of BK channels at presynaptic sites in thalamic neurons projecting onto BC-L5PN, the lack of paxilline effects on A-type PSPs supports the absence of effects of presynaptic BK channels in the synapses we studied. Two additional lines of evidence suggest a dominant role for postsynaptic NMDA-BK complexes: the modification in PSP kinetics after paxilline application exclusively in B-type neurons (Fig. 5) and the selective effect of intracellular paxilline on postsynaptic plasticity in B-type neurons (Fig. 6).

The interpretation of our results must take into account the possible influence of other coupling mechanisms involving BK and additional ion channels at dendrites. For instance, our experiments do not rule out the possibility that dendritic VGCC (61) or NMDAR-uncoupled BK channels may partially contribute to modulate the observed effects. Interestingly, a recent study suggests that SK-mediated inhibition of NMDARs is a general mechanism that regulates synaptic plasticity associated with BC-L5PN to BC-L5PN communication (62). This backward regulatory mechanism affects intralayer communication between regular-spiking BC-L5PNs and depends on back-propagating action potentials rather than NMDA activation. Although we cannot exclude a contribution from this process, there are three lines of evidence to suggest that the majority of effects we describe are due to NMDAR–BK coupling: 1) NMDAR activation resulted only in BK channel–mediated current, 2) BK-specific inhibition completely abolished the t-LTP differences between A-type and B-type BC-L5PNs, and 3) BK-mediated inhibition of NMDARs remained when basal afferent inputs were electrically stimulated and was independent of back-propagating action potentials. Therefore, although two different mechanisms for the inhibition of NMDARs may be present in the basal dendrites of BC-L5PNs, our data strongly suggest that NMDAR–BK modulation of synaptic transmission and long-term synaptic plasticity is a forward regulatory mechanism involving thalamocortical projections to a restricted population of BC-L5PNs in conditions in which the prior activation of postsynaptic NMDARs is mandatory, and action potentials are not required.

We used a low-frequency STDP protocol to induce LTP in our study. Under these experimental conditions, it has been proposed that GluN1/GluN2B NMDARs make a larger contribution to the total charge transfer than GluN1/GluN2A NMDARs (63) as expected from the slower deactivation rates of GluN2B-containing NMDARs (35). Taking this into account, GluN2B-containing NMDARs should conduct more Ca^2+^ than GluN1/GluN2A channels in our experimental conditions, activating BK more efficiently. That being the case, Ca^2+^ entry through GluN2B-containing NMDARs would be the main contributor to BK activation and thus the inhibitory mechanism underlying the modulation of synaptic transmission and LTP. This notion corresponds with our heterologous expression experiments using physiological concentrations of extracellular Na^+^ in which GluN1/GluN2B NMDARs produced a larger leftward shift in the BK activation curve than GluN1/GluN2A. It also correlates with our observations in basal dendrites of BC-L5PNs, where a specific blockade of GluN2B-containing NMDARs produced a larger reduction in the NMDA-evoked outward current. In summary, we have demonstrated that GluN2A- and GluN2B-containing NMDARs are able to activate BK channels in both heterologous expression systems and the basal dendrites of BC-L5PNs. Whether B-type neurons express a combination of GluN1/GluN2A and GluN1/GluN2B heteromers, or GluN1/GluN2A/GluN2B triheteromers (8), requires further investigation.

An interesting question arises from the observation that functional NMDAR–BK association is exclusive to the basal dendrites of a subpopulation of regular-spiking BC-L5PNs: Is it associated with the specific expression of particular GluN2 subunits? Although the role of GluN2C and GluN2D subunits was not investigated, a differential distribution of these and other subunits between A-type and B-type neurons would be reflected in the macroscopic NMDAR conductance, which is similar in both A-type and B-type BC-L5PNs. Therefore, we believe that both populations of BC-L5PNs exhibit a similar distribution of GluN2 subunits. Why, then, do NMDAR–BK associations occur only in the basal dendrites of B-type neurons? As both cell types express BK channels and NMDARs, a plausible explanation is that B-type neurons exhibit a distinct mechanism to target specific channels to dendritic compartments. This could be achieved by engaging scaffolding proteins, such as the receptor for activated C kinase 1 (RACK1) and caveolin-1, which are known to bind both the GluN2B NMDAR subunit (64, 65) and BK channels (66, 67). In addition, we observed a larger BK current in B-type than in A-type neurons, suggesting a higher abundance of BK channels in their membrane. Therefore, it is tempting to speculate that the formation of complexes and their targeting to the basal dendrites of B-type neurons depends on the overall abundance of BK channels and thus their availability to couple to NMDARs.

**Fig. 8.**
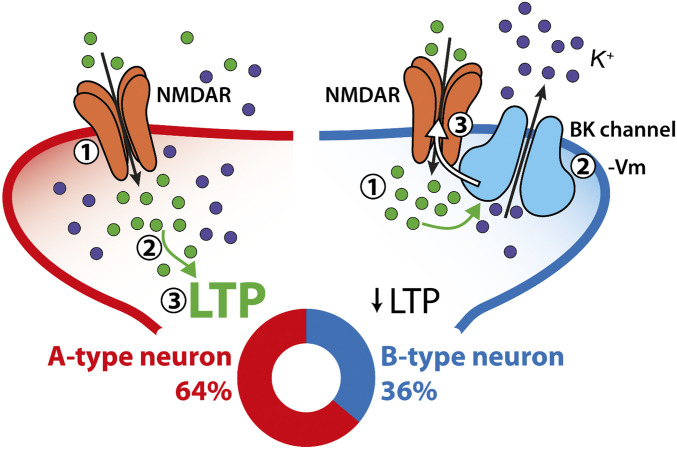
NMDAR–BK coupling controls dendrite-specific synaptic plasticity. Two populations of regular-spiking BC-L5PNs can be distinguished by the absence (A-type, *Left*, red) or presence (B-type, *Right*, blue) of NMDAR–BK functional association in basal dendrites. In B-type neurons (∼36%), NMDAR–BK coupling provides a negative feedback mechanism whereby the entry of Ca^2+^ associated with NMDAR activation (*1*) opens neighboring BK channels (*2*) that allow outward flow of K^+^. The resultant membrane hyperpolarization (−Vm) reinstates voltage-dependent Mg^2+^ block of NMDARs (*3*), truncating Ca^2+^ entry and increasing the threshold for long-term synaptic plasticity (↓LTP). We show in this study that B-type BC-L5PNs exhibit a higher threshold for the induction of t-LTP. On the other hand, A-type neurons (∼64%) lacking the NMDAR–BK molecular break undergo LTP (*3*) associated with Ca^2+^ entry (*2*) via NMDARs (*1*). Our data reveal that A-type neurons reach saturation at a lower stimulation frequency than B-type, independent of the number of synaptic inputs.

In this study, we uncover two populations of regular-spiking BC-L5PNs that are distinguished by the absence (A-type, ∼64%) or presence (B-type, ∼36%) of NMDAR–BK functional coupling in basal dendrites. Interestingly, this distribution resembles the electrophysiological characteristics and neuronal ratio of two previously described populations of mouse and rat BC-L5PNs, which were classified according to the presence or absence of a Ca^2+^ spike (24, 46, 47). Functional NMDAR–BK coupling is exclusive to the basal dendrites of B-type neurons (Fig. 8), which exhibit a higher threshold for the induction of LTP because of the BK-dependent inhibition of NMDARs. A-type neurons lack this molecular brake and therefore reach saturation at a lower stimulation frequency, independent of the number of synaptic inputs. This leads us to propose that BK-dependent inhibition of NMDARs endows B-type neurons with a calibration mechanism that allows them to decode the number and frequency of afferent synaptic inputs using selective synaptic plasticity attenuation. As a result of this discrimination capability, we hypothesize that the basal dendrites of B-type BC-L5PNs function as high-pass filters of thalamic afferent inputs, displaying the same output as A-type neurons when a strong stimulus or series of stimuli reach the dendrites (such as during high levels of pre- and postsynaptic coincident activity) but attenuating signals below a specific stimulation threshold. This cutoff would be mainly determined by the number of BK channels that are available to functionally couple to NMDARs in the basal dendrites of B-type neurons: the larger the number of available BK channels, the higher the threshold for the induction of synaptic plasticity. This mechanism would provide B-type BC-L5PNs with a dynamic range of output responses for the same afferent input stimuli, thus increasing the computational power of the somatosensory cortex.

In summary, we have demonstrated that the functional coupling of NMDARs and BK channels in the basal dendrites of a specific set of BC-L5PNs modulates synaptic transmission and synaptic plasticity in thalamocortical circuits. This finding unmasks the critical influence that the functional association of ion channels can have on the integration of afferent synaptic inputs by neurons.

## Methods

### Animal Procedures and Brain Slices Preparation.

All experimental procedures were approved by and carried out in accordance with University de La Laguna (ULL) Ethics Committee, Spanish (RD 53/2013) and European Commission (2010/63/EU) animal care guidelines. C57BL/6J mice (The Jackson Laboratory no. 000664) were housed in standard laboratory cages with ad libitum access to water and food in temperature- and humidity-controlled rooms under a 12:12 h light–dark cycle at the ULL animal facilities (registry no. ES380230024514). C57BL/6J adult male mice (postnatal 28 36) were slightly anesthetized with isoflurane, decapitated, and the brain quickly removed and immersed in an ice-cold high-sucrose solution (in mM: 189 sucrose, 10 glucose, 26 NaHCO_3_, 3 KCl, 5 MgSO_4_, 0.1 CaCl_2_, and 1.25 NaH_2_PO_4_) continuously bubbled with carbogen (95% oxygen/5% carbon dioxide). Coronal brain slices (350 µm) were obtained using a Microm HM650V vibratome (Thermo Scientific). Slices were maintained for 1 to 1.5 h in ACSF (in mM: 124 NaCl, 2.69 KCl, 1.25 KH_2_PO_4_, 2 MgSO_4_, 26 NaHCO_3_, 10 glucose, 2 CaCl_2_, and 0.4 ascorbic acid; pH 7.35) bubbled with carbogen at room temperature (22 to 24 °C).

### Electrophysiology in Brain Slices

Electrophysiological recordings in brain slices were performed using the whole-cell configuration of the patch-clamp technique (68) in a recording chamber mounted onto an upright Olympus BX51WI microscope (Olympus) where they were continuously perfused with carbogen-bubbled ACSF. Patch pipettes were made from 1.50 outer diameter (OD)/0.86 inner diameter (ID) borosilicate glass capillaries (GC150F-10; Harvard Apparatus) using a P-97 micropipette puller (Sutter Instruments) and had resistances of 5 to 8 MΩ when filled with the internal solution (in mM: 135 KMeSO_4_, 10 KCl, 10 Hepes, 5 NaCl, 2.5 ATP-Mg, and 0.3 GTP-Na; pH 7.3). Series resistance was compensated (∼70%), and recordings were discarded when the access resistance varied >20% during the experiment. Data were acquired at 10 kHz and low-pass filtered at 4 kHz with a MultiClamp 700A amplifier (Molecular Devices) through an Axon Digidata 1550B interface board (Molecular Devices) controlled by pClamp software (Molecular Devices). All experiments were performed at room temperature (22 to 24 °C). Pyramidal neurons from layer 5 of the somatosensory 1 barrel field area were visualized by infrared microscopy and differential interference contrast using an ORCA-Flash4.0 LT digital camera (Hamamatsu). BC-L5PN were identified by their location (just below layer 4), shape (characteristic somatic morphology), and electrophysiological properties (only regular-spiking neurons were used in the study; intrinsically bursting and fast-spiking neurons were discarded), suggesting that we predominantly recorded from slender-tufted neurons located in layer 5a as described previously (50, 51).

Currents evoked by NMDA application at different holding potentials (from −60 to 0 mV) were recorded from BC-L5PN using the voltage-clamp mode in the whole-cell configuration of the patch-clamp technique in Mg^2+^-free ACSF (by replacing Mg^2+^ with Ca^2+^) supplemented with TTX (1 µM) and NMDAR coagonist glycine (10 µM). NMDA (200 μM) was locally delivered (puff application) at different dendritic locations of BC-L5PN through a glass pipette (GC150F-10 borosilicate glass capillaries) using a PMI-100 Pressure Microinjector (Dagan Corporation) at 1 bar during 50 to 200 ms. The duration of the NMDA puff application was adjusted to obtain inward currents of 200 to 250 pA peak amplitude at −60 mV. NMDA-evoked inward currents (I_Inward_) were measured as the maximum peak amplitude. NMDA-evoked inward and outward total ionic charges (Q_Inward_, Q_Outward_) were calculated as the area under the trace. In the experiments in which BAPTA (15 or 1 mM) and EGTA (15 mM) Ca^2+^ chelators were added to the recording pipette, KMeSO_4_ was equimolarly replaced by BAPTA-K or EGTA-K to maintain the total internal K^+^ concentration.

BC-L5PN action potentials were recorded using the current-clamp mode in the whole-cell configuration of the patch-clamp technique in normal ACSF (including 2 mM MgSO_4_ and 2 mM CaCl_2_) as indicated in the figure legends. The resting membrane potential, action potential firing, and cell input resistance were calculated from these recordings. Individual action potentials were evoked either by presynaptic electrical stimulation of basal afferent inputs or by a brief current injection through the recording pipette (5 ms, 200 to 400 pA). No differences between both methods were observed (*SI Appendix*, Fig. S5*A*). Action potential characteristics were calculated from these recordings (*SI Appendix*, Fig. S5).

PSPs were obtained by presynaptic electrical stimulation of basal afferent inputs in the limit between layers 5 and 6 (50, 51). Stimulation pipettes were made from 1.50 OD/1.02 ID thick septum theta borosilicate glass capillaries (TST150-6; World Precision Instruments) using a P-97 micropipette puller and filled with ACSF. Single pulses (100 µs) were delivered at 0.20 Hz by a Master-8 Pulse Stimulator (A.M.P.I.) through an ISU-165 isolation unit (Cibertec). Stimulus intensity was adjusted to obtain 3 to 5 mV PSP responses and was unchanged for the entire experiment. In those experiments in which the stimulus intensity was adjusted to evoke NMDAR-dependent Ca^2+^ spikes, voltage-gated Na^+^ channel blocker QX-314 (2 mM) was included in the recording pipette (30, 51). In all experiments, basal PSP were recorded for 10 to 15 min before any drug application.

The induction of STDP t-LTP was achieved by pairing pre- and postsynaptic action potentials (Δt = 10 ms). Presynaptic action potentials were evoked by electrical stimulation of basal afferent inputs as described above. Postsynaptic action potentials were elicited by brief current injection through the recording pipette (5 ms, 200 to 400 pA). The extent of PSP potentiation was evaluated by quantifying the PSP amplitude during 5 min before and 50 min after t-LTP induction protocols (30, 50, and 90 pairings). In Fig. 6*B*, paxilline (500 nM) was applied intracellularly through the recording pipette. In some experiments, presynaptic electrical stimulation was performed within the limit between layers 1 and 2 to stimulate the afferent inputs to BC-L5PNs apical dendrites.

### Electrophysiology in HEK293T Cells.

HEK293T cells (American Type Culture Collection no. CRL-3216) were grown on 12-mm polylysine-treated glass coverslips in Dulbecco’s Modified Eagle Medium supplemented with 10% fetal bovine serum and 1% penicillin-streptomycin and maintained in an incubator at 37 °C and 5% carbon dioxide atmosphere. Transient transfection was performed using jetPRIME (Polyplus Transfection) and different combinations of complementary DNA plasmids encoding for BK alpha subunit [pBNJ-hsloTAG (36)], GluN1 (pEYFP-NR1a; Addgene plasmid no. 17928), GluN2A (pEGFP-NR2A; Addgene plasmid no. 17924), and GluN2B (pEGFP-NR2B; Addgene plasmid no. 17925). All fluorescently tagged NMDAR plasmids were a gift from Stefano Vicini, Georgetown University School of Medicine, Washington, DC (69).

Electrophysiological recordings were performed 36 to 48 h posttransfection using the inside-out configuration of the patch-clamp technique on an inverted Nikon Eclipse Ti-U microscope (Nikon) coupled to an Axopatch-200B amplifier and a Digidata 1550A interface (Molecular Devices) (38, 68). All experiments were performed at room temperature (22 to 24 °C). Patch pipettes were fabricated from GC150F-10 borosilicate glass capillaries and fire polished with a MF-200 microforge (World Precision Instruments) to obtain a tip resistance of 2 to 5 MΩ when filled with the “extracellular” solution (in mM: 80 KMeSO_3_, 60 *N*-methylglucamine-MeSO_3_, 20 Hepes, 2 KCl, and 2 CaCl_2_; pH 7.4) supplemented with NMDA (200 µM) and glycine (10 µM) to ensure activation and significantly reduced desensitization of NMDAR during the experiment as previously described (70, 71). The bath (“intracellular”) solution contained (in mM) 80 KMeSO_3_, 60 *N*-methylglucamine-MeSO_3_, 20 Hepes, 2 KCl, and 1 hydroxyethylethylenediaminetriacetic acid (HEDTA) (pH 7.4). In recordings from patches expressing BK channels alone, CaCl_2_ was added to the bath solution to obtain the desired free Ca^2+^ concentration calculated using the MaxChelator program (72). Free Ca^2+^ was confirmed using a Ca^2+^-sensitive electrode (Thermolab Systems). Solutions containing 100 μM Ca^2+^ did not include the HEDTA chelator. Data were acquired at 100 kHz and low-pass filtered at 5 kHz. BK currents were elicited from a holding potential of −60 mV, stepping from −100 to 200 mV for 25 ms in 20 mV increments and then repolarizing to −80 mV for 100 ms. G–V curves were generated from tail current amplitudes normalized to the maximum amplitude obtained in 100 μM Ca^2+^. A Boltzmann equation was fitted to the data according to the equation

$$G\;/\;G_{max}=\;1\;/\;\left(1\;+\;exp\left(\left(V_{m}–\;V_{half}\right)\;/\;z\right)\right),$$

where *V*_*half*_ is the voltage of half-maximum activation, *z* is the slope of the curve, *V*_*m*_ is the test potential, and *G*_*max*_ is the maximal conductance.

In some experiments (Fig. 3 *F–H*), inside-out recordings were performed using slightly modified brain slices solutions to achieve physiological Na^+^ concentrations. Briefly, the pipette “extracellular” solution contained normal ACSF supplemented with NMDA (200 µM) and glycine (10 µM) to ensure NMDAR activation. The bath “intracellular” solution contained (in mM) 135 KMeSO_4_, 10 KCl, 10 Hepes, 5 NaCl, 2.5 ATP-Mg, and 0.3 GTP-Na (pH 7.3) and was supplemented with EGTA (10 mM) to obtain a Ca^2+^-free solution. BK currents were elicited from a holding potential of −60 mV, applying a family of pulses from −100 to +200 mV in 20 mV steps for 100 ms. In this case, G–V curves were generated from the current–voltage relationships (G = I/(V_m_ – E_rev_)), where I is the current amplitude at the end of the depolarizing pulse for each test potential (V_m_) and E_rev_ is the reversal potential for K^+^.

### PLA.

PLA was performed using the DuoLink Kit (Sigma-Aldrich). HEK293T cells expressing different combinations of NMDAR and BK channels were fixed with 4% paraformaldehyde for 20 min, permeabilized, and then blocked for 1 h at 37 °C to avoid nonspecific binding of antibodies. The BK channel was detected using a rabbit polyclonal anti-Maxi K^+^ channel alpha subunit primary antibody (1:200, no. ab219072; Abcam). GluN1, GluN2A, and GluN2B subunits of NMDAR were detected using goat polyclonal primary antibodies anti-NMDAR1 (1:200, no. NB100-41105; Novus Biologicals), mouse monoclonal anti-NMDAε1 (1:200, no. sc-515148; Santa Cruz Biotechnology), and anti-NMDAε2 (1:200, no. sc-365597; Santa Cruz Biotechnology), respectively. Secondary antibodies conjugated with oligonucleotides were supplied with the PLA DuoLink Kit. Controls consisted of untransfected HEK293T cells or cells expressing individually the BK alpha subunit or single NMDAR subunits. Image acquisition and analysis was performed using the Duolink Image Tool (Sigma-Aldrich) and Fiji (73) software on a Leica SP8 inverted confocal microscope (Leica Biosystems). The PLA technique allows the detection of protein–protein interactions (less than 40 nm) as quantifiable fluorescent dots (74). The results are expressed as the number of fluorescent signals per cell area (PLA signals/µm^2^).

### Drugs and Reagents.

D-AP5 (D-2-amino-5-phosphonovalerate, AP5), glycine, IFEN, NMDA, paxilline, QX-314, and TTX were purchased from Tocris. EGTA and HEDTA were purchased from Sigma-Aldrich. BAPTA and ZnCl_2_ were purchased from Abcam and Merck, respectively. Drugs were dissolved in the adequate solvent (dimethyl sulfoxide [DMSO] or water) and prepared as 1,000 to 10,000× stock solutions. A subsequent dilution in ACSF (bath application) or pipette recording solution was performed to obtain the final concentration used in the experiments.

### Statistical Analysis.

Data were analyzed with pClamp (Molecular Devices) and GraphPad Prism 8 (GraphPad). Data are shown as mean ± SEM or as individual values (symbols) plus the median and 25th to 75th percentile (boxes) values. Two-tailed Student’s *t* tests were used to analyze data from the same neuron measured before and after treatments (e.g., pharmacological characterization in Fig. 1*E* or t-LTP in Figs. 6 and 7). Two-tailed unpaired Student’s *t* tests (Gaussian distribution) or Mann–Whitney *U* tests (non-Gaussian distribution) were used to analyze A-type versus B-type BC-L5PN electrophysiological data. Kruskal–Wallis test (followed by Dunn’s tests) was used to analyze PLA data. Statistical significance is stated as *P < 0.05, **P < 0.01, or ***P < 0.001. Statistical details related to the main and supplementary figures are specified in *SI Appendix*, Table S1.

## Supplementary Material

Supplementary File

## Data Availability

All study data are included in the article and/or *SI Appendix*.
