# Supplementary material for: NMDA receptor–BK channel coupling regulates synaptic plasticity in the barrel cortex
Source: Proc Natl Acad Sci U S A. 2021 Aug 27;118(35):e2107026118. doi: 10.1073/pnas.2107026118 (PMC8536339; doi:10.1073/pnas.2107026118)
Supplement: Supplementary File [file pnas.2107026118.sapp.pdf]

PNAS
www.pnas.org

Supplementary Information for

NMDA receptor–BK channel coupling regulates synaptic plasticity in the barrel cortex

Ricardo Gómez, Laura E. Maglio, Alberto J. Gonzalez-Hernandez, Belinda Rivero- Pérez, David Bartolomé-Martín, and Teresa Giraldez

* Ricardo Gómez, *Teresa Giraldez

Email: *rgomezga@ull.edu.es *giraldez@ull.edu.es

This PDF file includes:

Figures S1 to S5
Table S1
Table S2
SI References

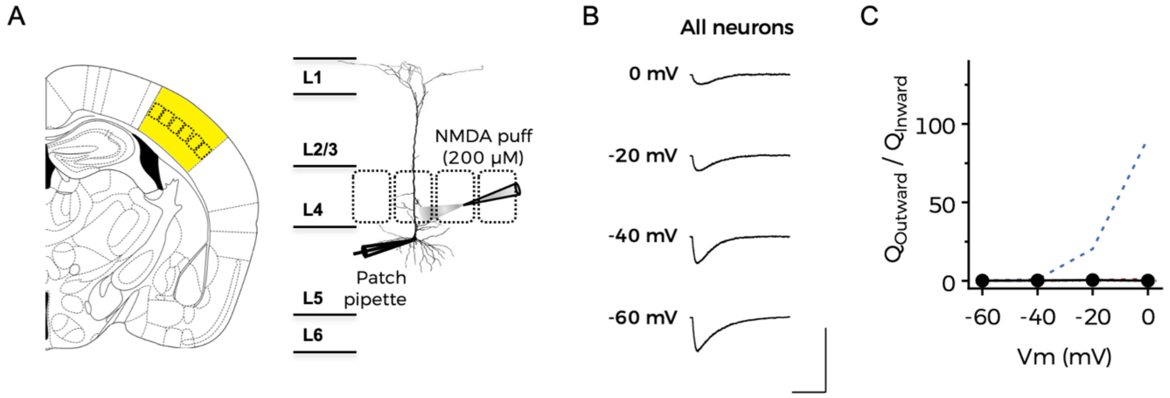

Fig. S1. NMDAR-dependent outward currents are absent in oblique dendrites and the initial segment of the apical dendrite. (A) Left, general representation view of a mouse brain slice with the barrel cortex area highlighted in yellow. Right, schematic representation of the experimental design. **(B)** Representative current traces obtained at the indicated holding potentials after NMDA application. Scale bars represent 10 s and 200 pA. **(C)** Average Q-V relationships. Data points represent mean ± SEM; n=12. Dashed lines represent data from Figure 1C for a better comparison.

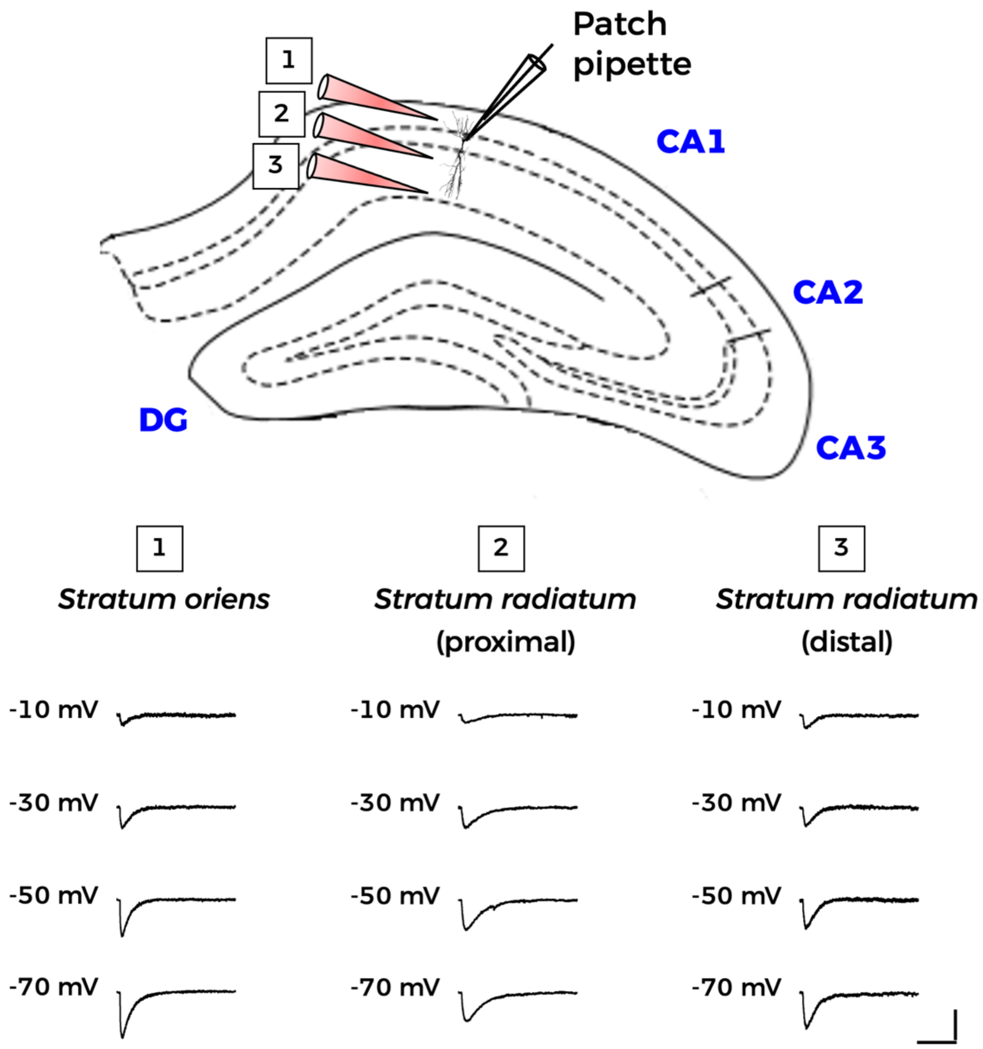

Fig. S2. NMDA-evoked currents in dendrites from hippocampal CA1 pyramidal neurons. Representative current traces obtained at the indicated holding potentials after NMDA application at different dendrite locations of hippocampal CA1 pyramidal neurons. No outward currents were observed in any case. Scale bars represent 10 s and 200 pA.

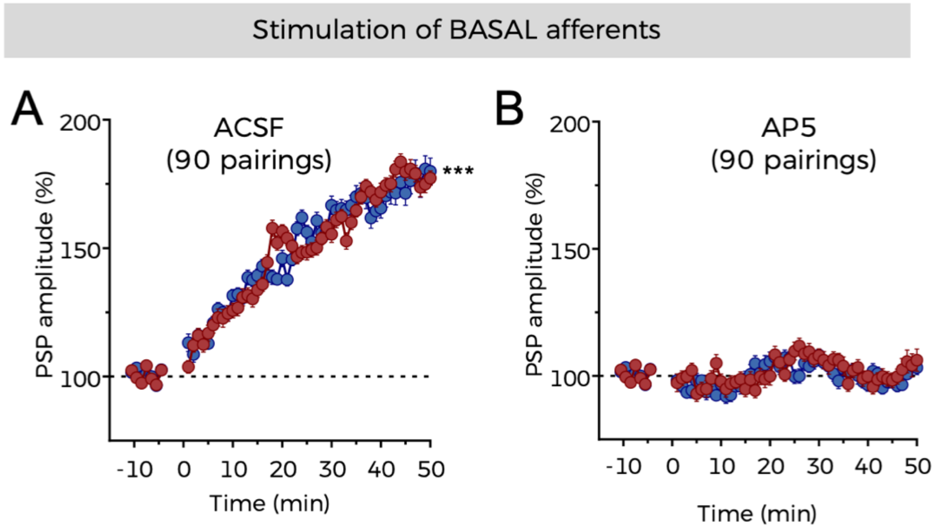

Fig. S3. NMDAR activation is mandatory for t-LTP induction. (A) Time course of t-LTP development over time in A-type (red) and B-type neurons (blue) in control conditions (ACSF), following the experimental design depicted in Figure 6A. **(B)** Same experiments as in panel **A** were performed in the presence of 100 µM AP5. Data points represent mean ± SEM. A-type (ACSF): n=5; B-type (ACSF): n=6; A-type (AP5): n=5; B-type (AP5) n=4. Data in panel **A** are the same as Figure 7C (right panel) and are shown here for a better comparison. In **A**, ***p<0.001 (t-LTP vs. basal conditions).

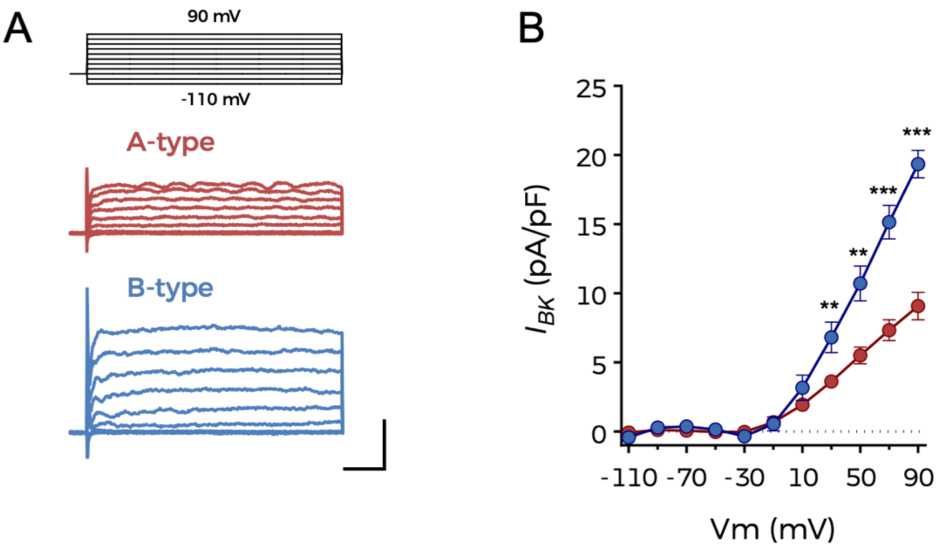

Fig. S4. BK channels are present in the plasma membrane of both types of BC-L5PN. (A) ***BK current recordings.*** Representative BK current traces obtained from an A-type (red) and a B-type neuron (blue) using the voltage protocol shown at the top and obtained as the paxilline-sensitive currents. BK currents from BC-L5PN were recorded as paxilline-sensitive currents using the voltage-clamp mode in the whole-cell configuration of the patch-clamp technique in normal ACSF (including 2 mM $MgSO_4$ and 2 mM $CaCl_2$) supplemented with TTX (1 µM). Patch pipettes were filled with a modified recording solution (in mM: 123 $KMeSO_3$, 9 NaCl, 9 HEPES, 0.9 EGTA, 14 Tris-phosphocreatine, 2 ATP-Mg, 2 ATP-Na, and 0.3 GTP-Tris; pH 7.3), as previously described (1). I-V relationships were elicited from a holding potential of -70 mV, stepping from -110 to +90 mV for 150 ms in 20 mV increments. BK currents were isolated by current subtraction after bath application of the BK blocker paxilline (1 µM) and normalized to BC-L5PN capacitance (1). **(B)** BK current density for A-type (red; n=8) and B-type (blue; n=4) neurons as a function of different membrane potentials. Data points represent mean ± SEM. In **B**, $^{**}p<0.01$ and $^{***}p<0.001$ (B-type vs. A-type).

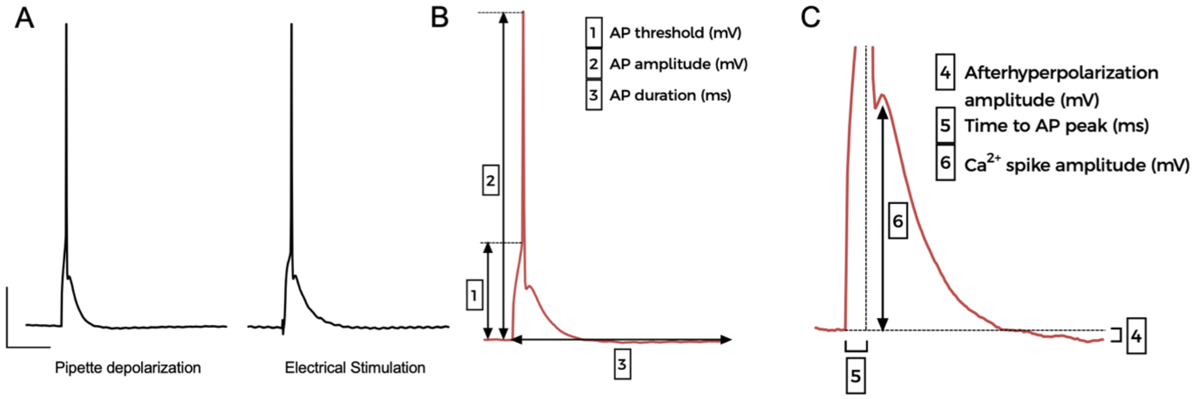

Fig. S5. Single action potential characteristics and measurements. (A) Representative single action potentials recorded from the same A-type BC-L5PN, evoked through pipette depolarization (left) or after electrical stimulation of basal afferent synaptic inputs (right). **(B)** Schematic description of the single action potential parameters determination summarized in Figure 4: threshold, amplitude, and total duration. **(C)** Determination of single action potential parameters summarized in Figure 4.

Table S1. Summary of statistical significance and tests used in each figure (comparisons reaching statistical significance are highlighted in light yellow)

Figure 1. NMDAR activation opens BK channels in BC-L5PN basal dendrites				
**PANEL**	**STATISTIC**	**CONDITION**	**P VALUE**	**OUTPUT**
Fig. 1C<br><br>Qoutward/<br>Qinward	Unpaired t-test<br>(two-tailed)	-60 mV (B vs. A)	0.1851	Not significant
		-40 mV (B vs. A)	0.0990	Not significant
		-20 mV (B vs. A)	0.0014	**
		0 mV (B vs. A)	0.0041	**
Fig. 1D<br><br>NMDAR<br>Current	Unpaired t-test<br>(two-tailed)	-60 mV (B vs. A)	0.9994	Not significant
		-40 mV (B vs. A)	0.6021	Not significant
		-20 mV (B vs. A)	0.9175	Not significant
		0 mV (B vs. A)	0.7962	Not significant
Fig. 1D<br><br>NMDAR<br>Charge	Unpaired t-test<br>(two-tailed)	-60 mV (B vs. A)	0.9997	Not significant
		-40 mV (B vs. A)	0.7394	Not significant
		-20 mV (B vs. A)	0.0449	*
		0 mV (B vs. A)	0.0435	*
Fig. 1E<br>(top)<br><br>Outward<br>component	Paired t-test<br>(two-tailed)	ACSF vs. AP5	<0.0001	***
		ACSF vs. Zn^{2+}	0.0002	***
		ACSF vs. Zn^{2+}+AP5	<0.0001	***
		Zn^{2+} vs. Zn^{2+}+AP5	0.0003	###
		ACSF vs. IFEN	<0.0001	***
		ACSF vs. IFEN+AP5	<0.0001	***
		IFEN vs. IFEN+AP5	0.0057	##
		ACSF vs. PAX	<0.0001	***
		ACSF vs. PAX+AP5	<0.0001	***
		PAX vs. PAX+AP5	0.5845	Not significant
Fig. 1E<br>(bottom)<br><br>Inward<br>component	Paired t-test<br>(two-tailed)	ACSF vs. AP5	<0.0001	***
		ACSF vs. Zn^{2+}	0.0781	Not significant
		ACSF vs. Zn^{2+}+AP5	0.0010	***
		Zn^{2+} vs. Zn^{2+}+AP5	0.0274	#
		ACSF vs. IFEN	0.0785	Not significant
		ACSF vs. IFEN+AP5	0.0040	**
		IFEN vs. IFEN+AP5	0.0413	#
		ACSF vs. PAX	0.0381	*
		ACSF vs. PAX+AP5	<0.0001	***
		PAX vs. PAX+AP5	0.0283	#

Figure 2. NMDARs and BK channels are within functional proximity in B-type BC-L5PNs				
**PANEL**	**STATISTIC**	**CONDITION**	**P VALUE**	**OUTPUT**
Fig. 2E<br>EGTA 15 mM	Unpaired t-test (two-tailed, multiple t-test)	-60 mV (B vs. A)	0.5963	Not significant
		-40 m V (B vs. A)	0.0541	Not significant
		-20 m V (B vs. A)	0.0040	**
		0 m V (B vs. A)	0.0310	*
Fig. 2F<br>BAPTA 1 mM	Unpaired t-test (two-tailed, multiple t-test)	-60 mV (B vs. A)	0.6245	Not significant
		-40 m V (B vs. A)	0.0020	**
		-20 m V (B vs. A)	0.0095	**
		0 m V (B vs. A)	0.0058	**

Figure 3. Both GluN2A- and GluN2B-containing NMDARs can functionally couple to BK channels				
**PANEL**	**STATISTIC**	**CONDITION**	**P VALUE**	**OUTPUT**
Fig. 3B<br>PLA	Kruskal-Wallis test (followed by Dunn's tests)	BK+GluN1/GluN2A vs. UNTRANSF, BK, and GluN1	<0.0001	***
		BK+GluN1/GluN2B vs. UNTRANSF, BK, and GluN1	<0.0001	***
Fig. 3E<br>V_{half}<br>Symm. K^+	Mann-Whitney U test (two-tailed)	BK+2A vs. BK (0 Ca^{2+})	<0.0001	***
		BK+2A vs. BK (1 Ca^{2+})	<0.0001	***
		BK+2A vs. BK (10 Ca^{2+})	0.6165	Not significant
		BK+2A vs. BK (100 Ca^{2+})	0.0032	**
		BK+2B vs. BK (0 Ca^{2+})	0.0003	***
		BK+2B vs. BK (1 Ca^{2+})	0.0003	***
		BK+2B vs. BK (10 Ca^{2+})	0.5743	Not significant
		BK+2B vs. BK (100 Ca^{2+})	0.0023	**
		BK+2A vs. BK+2B	0.5368	Not significant
Fig. 3H<br>V_{half}<br>Slices sol.	Mann-Whitney U test (two-tailed)	BK+2A vs. BK (0 Ca^{2+})	0.0008	***
		BK+2B vs. BK (0 Ca^{2+})	0.0005	***

Figure 4. A subpopulation of regular-spiking BC-L5PNs exhibit NMDAR–BK functional coupling				
**PANEL**	**STATISTIC**	**CONDITION**	**P VALUE**	**OUTPUT**
Fig. 4D	Mann-Whitney U test (two-tailed)<br>B-type vs. A-type	Resting membrane potential	0.4595	Not significant
Fig. 4E		Input resistance	0.2386	Not significant
Fig. 4F		Capacitance	0.8766	Not significant
Fig. 4G		Action potential freq. (200 pA)	0.4088	Not significant
		Action potential freq. (230 pA)	0.6359	Not significant
		Action potential freq. (260 pA)	0.8089	Not significant
		Action potential freq. (290 pA)	0.8481	Not significant
		Action potential freq. (320 pA)	0.8815	Not significant
Fig. 4H		Action potential threshold	0.2285	Not significant
Fig. 4I		Action potential amplitude	0.6334	Not significant
Fig. 4J		Action potential duration	<0.0001	***
Fig. 4K		I_{AHP} amplitude	<0.0001	***

Figure 5. BK-dependent inhibition of NMDARs reduces postsynaptic response amplitude				
**PANEL**	**STATISTIC**	**CONDITION**	**P VALUE**	**OUTPUT**
Fig. 5B PSP Amplitude	Paired t-test (two-tailed)	A-type: ACSF vs. PAX	0.0631	Not significant
		A-type: ACSF vs. PAX+AP5	0.0004	***
		A-type: PAX vs. PAX+AP5	0.0003	***
		B-type: ACSF vs. PAX	0.0021	**
		B-type: ACSF vs. PAX+AP5	0.0027	**
		B-type: PAX vs. PAX+AP5	0.0004	***
Fig. 5B PSP Area	Paired t-test (two-tailed)	A-type: ACSF vs. PAX	0.8007	Not significant
		A-type: ACSF vs. PAX+AP5	0.0018	**
		A-type: PAX vs. PAX+AP5	0.0179	*
		B-type: ACSF vs. PAX	0.0029	**
		B-type: ACSF vs. PAX+AP5	0.0010	**
		B-type: PAX vs. PAX+AP5	0.0021	**
Fig. 5B Rise Time	Paired t-test (two-tailed)	A-type: ACSF vs. PAX	0.7043	Not significant
		A-type: ACSF vs. PAX+AP5	0.0785	Not significant
		A-type: PAX vs. PAX+AP5	0.3442	Not significant
		B-type: ACSF vs. PAX	0.0008	***
		B-type: ACSF vs. PAX+AP5	0.0014	**
		B-type: PAX vs. PAX+AP5	<0.0001	***
Fig. 5B Decay Time	Paired t-test (two-tailed)	A-type: ACSF vs. PAX	0.4604	Not significant
		A-type: ACSF vs. PAX+AP5	0.0028	**
		A-type: PAX vs. PAX+AP5	0.0076	**
		B-type: ACSF vs. PAX	0.0004	***
		B-type: ACSF vs. PAX+AP5	0.0030	**
		B-type: PAX vs. PAX+AP5	<0.0001	***
Fig. 5D PSP Amplitude	Paired t-test (two-tailed)	A-type: ACSF vs. PAX	0.3446	Not significant
		A-type: ACSF vs. PAX+AP5	0.0011	**
		A-type: PAX vs. PAX+AP5	0.0102	*
		B-type: ACSF vs. PAX	0.0015	**
		B-type: ACSF vs. PAX+AP5	0.0011	**
		B-type: PAX vs. PAX+AP5	0.0003	***
Fig. 5D PSP Area	Paired t-test (two-tailed)	A-type: ACSF vs. PAX	0.1448	Not significant
		A-type: ACSF vs. PAX+AP5	0.0013	**
		A-type: PAX vs. PAX+AP5	0.0158	*
		B-type: ACSF vs. PAX	0.0092	**
		B-type: ACSF vs. PAX+AP5	0.0073	**
		B-type: PAX vs. PAX+AP5	0.0040	**
Fig. 5D Rise Time	Paired t-test (two-tailed)	A-type: ACSF vs. PAX	0.0518	Not significant
		A-type: ACSF vs. PAX+AP5	0.4969	Not significant
		A-type: PAX vs. PAX+AP5	0.8170	Not significant
		B-type: ACSF vs. PAX	0.0303	*
		B-type: ACSF vs. PAX+AP5	0.5845	Not significant
		B-type: PAX vs. PAX+AP5	0.0237	*
Fig. 5D Decay Time	Paired t-test (two-tailed)	A-type: ACSF vs. PAX	0.4425	Not significant
		A-type: ACSF vs. PAX+AP5	0.0124	*
		A-type: PAX vs. PAX+AP5	0.0648	Not significant
		B-type: ACSF vs. PAX	0.0394	*
		B-type: ACSF vs. PAX+AP5	0.0339	*
		B-type: PAX vs. PAX+AP5	0.0047	**

Figure 6. NMDAR–BK coupling increases the threshold for induction of synaptic plasticity				
**PANEL**	**STATISTIC**	**CONDITION**	**P VALUE**	**OUTPUT**
Fig. 6B<br><br>Basal afferents (ACSF)	Paired t-test (two-tailed)	A-type (30 p) Basal vs. t-LTP	<0.0001	***
		B-type (30 p) Basal vs. t-LTP	0.4115	Not significant
	Unpaired t-test (two-tailed)	B-type vs. A-type (30 p) t-LTP	<0.0001	###
Fig. 6B<br><br>Basal afferents (PAX_{int})	Paired t-test (two-tailed)	A-type (30 p) Basal vs. t-LTP	<0.0001	***
		B-type (30 p) Basal vs. t-LTP	<0.0001	***
	Unpaired t-test (two-tailed)	B-type vs. A-type (30 p) t-LTP	0.9336	Not significant
Fig. 6D<br><br>Apical afferents (ACSF)	Paired t-test (two-tailed)	A-type (30 p) Basal vs. t-LTP	0.3306	Not significant
		A-type (90 p) Basal vs. t-LTP	<0.0001	***
		B-type (90 p) Basal vs. t-LTP	<0.0001	***
	Unpaired t-test (two-tailed)	B-type vs. A-type (90 p) t-LTP	0.7896	Not significant

Figure 7. A high number and frequency of pre-post pairings relieves BK-dependent NMDAR inhibition				
**PANEL**	**STATISTIC**	**CONDITION**	**P VALUE**	**OUTPUT**
Fig. 7A<br><br>t-LTP 0.20 Hz	Paired t-test (two-tailed)	A-type (30 p) Basal vs. t-LTP	<0.0001	***
		B-type (30 p) Basal vs. t-LTP	0.4115	Not significant
		A-type (50 p) Basal vs. t-LTP	<0.0001	***
		B-type (50 p) Basal vs. t-LTP	<0.0001	***
		A-type (90 p) Basal vs. t-LTP	<0.0001	***
		B-type (90 p) Basal vs. t-LTP	<0.0001	***
Fig. 7B<br><br>Summary 0.20 Hz	Unpaired t-test (two-tailed)	B-type vs. A-type (30 p) t-LTP	<0.0001	###
		B-type vs. A-type (50 p) t-LTP	<0.0001	###
		B-type vs. A-type (50 p) t-LTP	<0.0001	###
Fig. 7C<br><br>t-LTP 0.33 Hz	Paired t-test (two-tailed)	A-type (30 p) Basal vs. t-LTP	<0.0001	***
		B-type (30 p) Basal vs. t-LTP	<0.0001	***
		A-type (50 p) Basal vs. t-LTP	<0.0001	***
		B-type (50 p) Basal vs. t-LTP	<0.0001	***
		A-type (90 p) Basal vs. t-LTP	<0.0001	***
		B-type (90 p) Basal vs. t-LTP	<0.0001	***

**Figure 7 (continuation).<br>A high number and frequency of pre-post pairings relieves BK-dependent NMDAR inhibition**				
**PANEL**	**STATISTIC**	**CONDITION**	**P VALUE**	**OUTPUT**
Fig. 7D<br><br>Summary<br>0.33 Hz	Unpaired t-test<br>(two-tailed)	B-type vs. A-type (30 p)<br>t-LTP	<0.0001	###
		B-type vs. A-type (50 p)<br>t-LTP	<0.0001	###
		B-type vs. A-type (90 p)<br>t-LTP	0.4680	Not significant
**Supplementary Figure 3 (related to Figure 6).<br>NMDAR activation is mandatory for t-LTP induction**				
Fig. S2A<br><br>t-LTP<br>0.33 Hz<br>(ACSF)	Paired t-test<br>(two-tailed)	A-type (90 p)<br>Basal vs. t-LTP	<0.0001	***
		B-type (90 p)<br>Basal vs. t-LTP	<0.0001	***
	Unpaired t-test<br>(two-tailed)	B-type vs. A-type (90 p)<br>t-LTP	0.4680	Not significant
Fig. S2B<br><br>t-LTP<br>0.33 Hz<br>(AP5)	Paired t-test<br>(two-tailed)	A-type (90 p)<br>Basal vs. t-LTP	0.1026	Not significant
		B-type (90 p)<br>Basal vs. t-LTP	0.0821	Not significant
	Unpaired t-test<br>(two-tailed)	B-type vs. A-type (90 p)<br>t-LTP	0.4210	Not significant
**Supplementary Figure 4 (related to Figure 6).<br>BK channels are present in the plasma membrane of both types of BC-L5PN**				
Fig. S3B<br><br>BK current	Unpaired t-test<br>(two-tailed)	-10 mV (B vs. A)	0.8611	Not significant
		+10 mV (B vs. A)	0.1025	Not significant
		+30 mV (B vs. A)	0.0073	**
		+50 mV (B vs. A)	0.0012	**
		+70 mV (B vs. A)	0.0002	***
		+90 mV (B vs. A)	<0.0001	***

Supplementary Table 2.

KEY RESOURCES TABLE

REAGENT or RESOURCE	SOURCE	IDENTIFIER
**Antibodies**		
Rabbit polyclonal anti-Maxi potassium channel α (BK α subunit)	Abcam	Cat#ab219072
Goat polyclonal anti-NMDAR1 (GluN1 subunit)	Novus Biologicals	Cat#NB100-41105
Mouse monoclonal anti-NMDAε1 (GluN2A subunit)	Santa Cruz Biotechnology	Cat#sc-515148
Mouse monoclonal anti-NMDAε2 (GluN2B subunit)	Santa Cruz Biotechnology	Cat#sc-365597
**Chemicals, Peptides, and Recombinant Proteins**		
D-AP5 (D-2-amino-5-phosphonovalerate, AP5)	Tocris	Cat#0106
BAPTA (1,2-bis(o-aminophenoxy)ethane-N,N,N',N'-tetraacetic acid)	Abcam	Cat#ab144924
EGTA (Ethylene glycol-bis(β-aminoethyl ether)-N,N,N',N'-tetraacetic acid)	Sigma-Aldrich	Cat#E4378
Glycine	Tocris	Cat#0219
HEDTA (N-(2-Hydroxyethyl)ethylenediamine-N,N',N'-triacetic acid)	Sigma-Aldrich	Cat#H8126
Ifenprodil	Tocris	Cat#0545
NMDA (N-methyl-D-aspartate)	Tocris	Cat#0114
Paxilline	Tocris	Cat#2006
QX-314 (N-(2,6-Dimethylphenylcarbamoylmethyl)-triethylammonium bromide)	Tocris	Cat#1014
TTX (Tetrodotoxin)	Tocris	Cat#1078
$ZnCl_2$ (Zinc chloride)	Merck	Cat#1.08816.1000
**Critical Commercial Assays**		
PLA: DuoLink In Situ Detection Reagents Red	Sigma-Aldrich	Cat#DUO92008
PLA: DuoLink In Situ PLA Probe Anti-Rabbit PLUS Antibody	Sigma-Aldrich	Cat#DUO92002; RRID:AB_10950581
PLA: DuoLink In Situ PLA Probe Anti-Mouse MINUS Antibody	Sigma-Aldrich	Cat#DUO92004; RRID:AB_2713942
PLA: DuoLink In Situ PLA Probe Anti-Goat MINUS Antibody	Sigma-Aldrich	Cat#DUO92006; RRID:AB_10953178
Cell transfection: jetPRIME reagent	Polyplus transfection	Cat#114-75
**Experimental Models: Cell Lines**		
Human: HEK293T cells	ATCC	Cat#CRL-3216
**Experimental Models: Organisms/Strains**		
Mouse: C57BL/6J	The Jackson Laboratory	Cat#000664; RRID:IMSR_JAX:000664
**Recombinant DNA**		
Plasmid: pBNJ-hsloTAG	(2)	N/A
Plasmid: pEYFP-NR1a	(3)	RRID: Addgene_17928
Plasmid: pEGFP-NR2A	(3)	RRID: Addgene_17924
Plasmid: pEGFP-NR2B	(3)	RRID: Addgene_17925
**Software and Algorithms**		

pClamp software package (Clampex, Clampfit)	Molecular Devices	https://www.moleculardevices.com/products/axon-patch-clamp-system/acquisition-and-analysis-software/pclamp-software-suite RRID:SCR_011323
MaxChelator	(4)	https://somapp.ucdmc.ucdavis.edu/pharmacology/bers/maxchelator/index.html
Prism8	GraphPad Inc.	https://www.graphpad.com/scientific-software/prism/ RRID:SCR_002798
Fiji	(5)	http://fiji.sc RRID:SCR_002285

SI References

1. J. P. Whitt, B. A. McNally, A. L. Meredith, Differential contribution of Ca(2+) sources to day and night BK current activation in the circadian clock. *The Journal of general physiology* **150**, 259-275 (2018).
2. T. Giraldez, T. E. Hughes, F. J. Sigworth, Generation of functional fluorescent BK channels by random insertion of GFP variants. *The Journal of general physiology* **126**, 429-438 (2005).
3. J. H. Luo *et al.*, Functional expression of distinct NMDA channel subunits tagged with green fluorescent protein in hippocampal neurons in culture. *Neuropharmacology* **42**, 306-318 (2002).
4. D. M. Bers, C. W. Patton, R. Nuccitelli, A practical guide to the preparation of Ca(2+) buffers. *Methods Cell Biol* **99**, 1-26 (2010).
5. J. Schindelin *et al.*, Fiji: an open-source platform for biological-image analysis. *Nat Methods* **9**, 676-682 (2012).
